# Integration and validation of complementary ex vivo assays for functional precision oncology

**DOI:** 10.1038/s41698-026-01555-2

**Published:** 2026-06-17

**Authors:** Anna Loboda, Jasmina Paluncic, Micaela Freitas, Marko Weidmann, Sonja Herter, Constantia Zeiser, Juliana P. Schulz, Attila Jády, Mirjam Blattner-Johnson, Robert J. Autry, Karen Frese, Dmitry Lupar, Martin Schneider, Roland Imle, Ana Banito, Hanno Glimm, Olaf Witt, Heike Peterziel, Jens M. Kelm, Claudia R. Ball, Ina Oehme

**Affiliations:** 1 Hopp Children’s Cancer Center Heidelberg (KiTZ), Heidelberg, Germany; 2 National Center for Tumor Diseases (NCT), NCT Heidelberg, a Partnership Between DKFZ and Heidelberg University Hospital, Heidelberg, Germany; 3 Clinical Cooperation Unit Pediatric Oncology, German Cancer Research Center (DKFZ) and German Cancer Consortium (DKTK), Heidelberg, Germany; 4 Faculty of Biosciences, Heidelberg University, Heidelberg, Germany; 5 Department for Translational Medical Oncology, National Center for Tumor Diseases Dresden (NCT/UCC), a partnership between DKFZ, Faculty of Medicine and University Hospital Carl Gustav Carus, TUD Dresden University of Technology, and Helmholtz-Zentrum Dresden—Rossendorf (HZDR), Dresden, Germany; 6 Translational Medical Oncology, Faculty of Medicine and University Hospital Carl Gustav Carus, TUD Dresden University of Technology, Dresden, Germany; 7 PreComb AG, Hombrechtikon, Switzerland; 8 Faculty of Medicine, Heidelberg University, Heidelberg, Germany; 9 Division of Pediatric Glioma Research, German Cancer Research Center (DKFZ), Heidelberg, Germany; 10 Division of Pediatric Neurooncology, German Cancer Research Center (DKFZ), Heidelberg, Germany; 11 German Center for Cardiovascular Research (DZHK), partner site Heidelberg, Heidelberg, Germany; 12 Soft-tissue sarcoma research group, German Cancer Research Center (DKFZ), Heidelberg, Germany; 13 Department of General, Visceral and Transplantation Surgery, Heidelberg University Hospital, Heidelberg, Germany; 14 Division of Pediatric Neurooncology, German Cancer Consortium (DKTK) and German Cancer Research Center (DKFZ), Heidelberg, Germany; 15 Department of Pediatric Oncology, Hematology, Immunology and Pulmonology, Heidelberg University Hospital, Heidelberg, Germany; 16 Translational Functional Cancer Genomics, National Center for Tumor Diseases (NCT) and German Cancer Research Center (DKFZ), Heidelberg, Germany; 17 German Cancer Consortium (DKTK), partner site Dresden, Dresden, Germany; 18 Faculty of Biology, TUD Dresden University of Technology, Dresden, Germany

**Keywords:** Cancer, Drug discovery, Oncology

## Abstract

Functional precision oncology (FPO) enables individualized therapy selection using patient-derived tumor models, yet the concordance of distinct ex vivo testing strategies remains unclear. Here, we compare two orthogonal drug sensitivity platforms across molecularly characterized models spanning diverse pediatric (*n* = 13) and adult (*n* = 6) cancers. A rapid ATP-based assay quantifies viability within three days, whereas a long-term dynamic image-based platform captures microtumor dynamics over two weeks, incorporating pharmacokinetic features. Up to 50 drugs were profiled across both platforms, with additional combinations evaluated in the long-term assay; genomics-guided targeted drugs served as benchmarks. Both approaches robustly distinguished responders from non-responders and showed strong agreement in therapeutic prioritization (96.5% within 95% limits of agreement). The long-term dynamic platform achieved 81% sensitivity and 78% specificity, while resolving response depth and distinguishing cytostatic from cytotoxic effects. A representative sarcoma case highlights clinical relevance: long-term dynamic profiling predicted disease progression, whereas the short-term assay captured early treatment-associated viability effects. These findings establish cross-platform reproducibility in FPO and provide a systematic benchmarking of such approaches. Defining their complementary utility will be essential for integrating FPO strategies into clinical decision-making.

## Introduction

In precision oncology, therapeutic decision-making is primarily guided by well-validated strategies that assess immunohistological and genetic features of the tumor. These diagnosis and genomic-driven approaches have demonstrated significant clinical utility in identifying actionable targets. However, there is growing interest and effort in integrating additional layers of functional information to further refine therapy recommendations. One such strategy involves testing drug response directly in patient tumor cells ex vivo in cell culture, providing complementary evidence to genomic data^1^. Functional precision oncology (FPO) has emerged as a promising approach to address limitations, such as capturing the dynamic and context dependent interactions between tumors and drugs^1^. By evaluating patient-specific tumor cell responses to drugs, it offers supplementary insights into tumor vulnerabilities and can further support or even expand genomic-driven stratification by providing the physician with a menu of therapeutic options to choose from. To overcome the limitations of static features, there has been a substantial progress in the field of drug testing platforms. These platforms now allow for the testing of drugs and drug combinations on patient-specific viable cancer cells^1^. These platforms encompass a wide range of tumor cell models, including (i) single cell suspensions^2^, (ii) monolayers^3,4^, (iii) cell-line based spheroids^5^, (iv) organoids^6^, (v) microtumors^7^ and (vi) PDX^8^. Across these diverse models, current practice is dominated by short-term viability assays, providing only static endpoints after 3–4 days of continuous drug exposure. Dynamic treatment monitoring has traditionally been performed in mouse xenograft models, which allow longitudinal readouts, but have limitations in scalability, precise human-relevant pharmacokinetic control and timeliness. More recently, emerging ex vivo dynamic assays, e.g., based on live-cell imaging or metabolic profiling are being developed to directly capture treatment kinetics in patient-derived models, though most remain in early translational stages^9^. Monitoring drug response kinetics provides complementary insights into drug efficacy, enabling the discrimination between responders and non-responders, and offering early insights on the potential clinical outcomes of responders. The high potential of FPO is highlighted by the growing number of precision oncology programs^10,11^ and clinical trials employing FPO strategies^1,12,13^. Various clinical questions are currently being addressed using the FPO methodologies^14^. The optimal platform for a given purpose may vary, dependent on the clinical context and the particular circumstances of the patient. Consequently, it would be erroneous to hypothesize the existence of a singular platform that encompasses all the diverse facets of clinical therapy decision-making. The wide range of FPO methodologies presents significant challenges in establishing the relative merits of each approach. Nevertheless, to advance the field of FPO, it is essential to systematically assess the performance and limitations of these diverse approaches and to clearly define their optimal applications. To enhance comprehension of platform performance and to facilitate more profound insight regarding the identification of responders and non-responders, a comparison was conducted between two platforms that are conceptually different. The comparison was performed regarding the following: a static cell viability assay^11^ and a dynamic growth kinetic-based platform. Both platforms were validated against a range of pediatric cancer cell models, encompassing various cancer types with defined driver alterations. From a validation standpoint, pediatric cancer cells may be advantageous compared to those from adult tumors, as children have accumulated fewer somatic mutations over time^15^. Furthermore, a range of on- and off-label drugs was tested, and the resulting data were compared. Finally, to complement the rare pediatric tumors with more common entities, cancer models derived from adult patients were also included in the study.

## Results

### Two independent drug-testing workflows allow for a systematic comparison of treatment responses

**Fig. 1:**
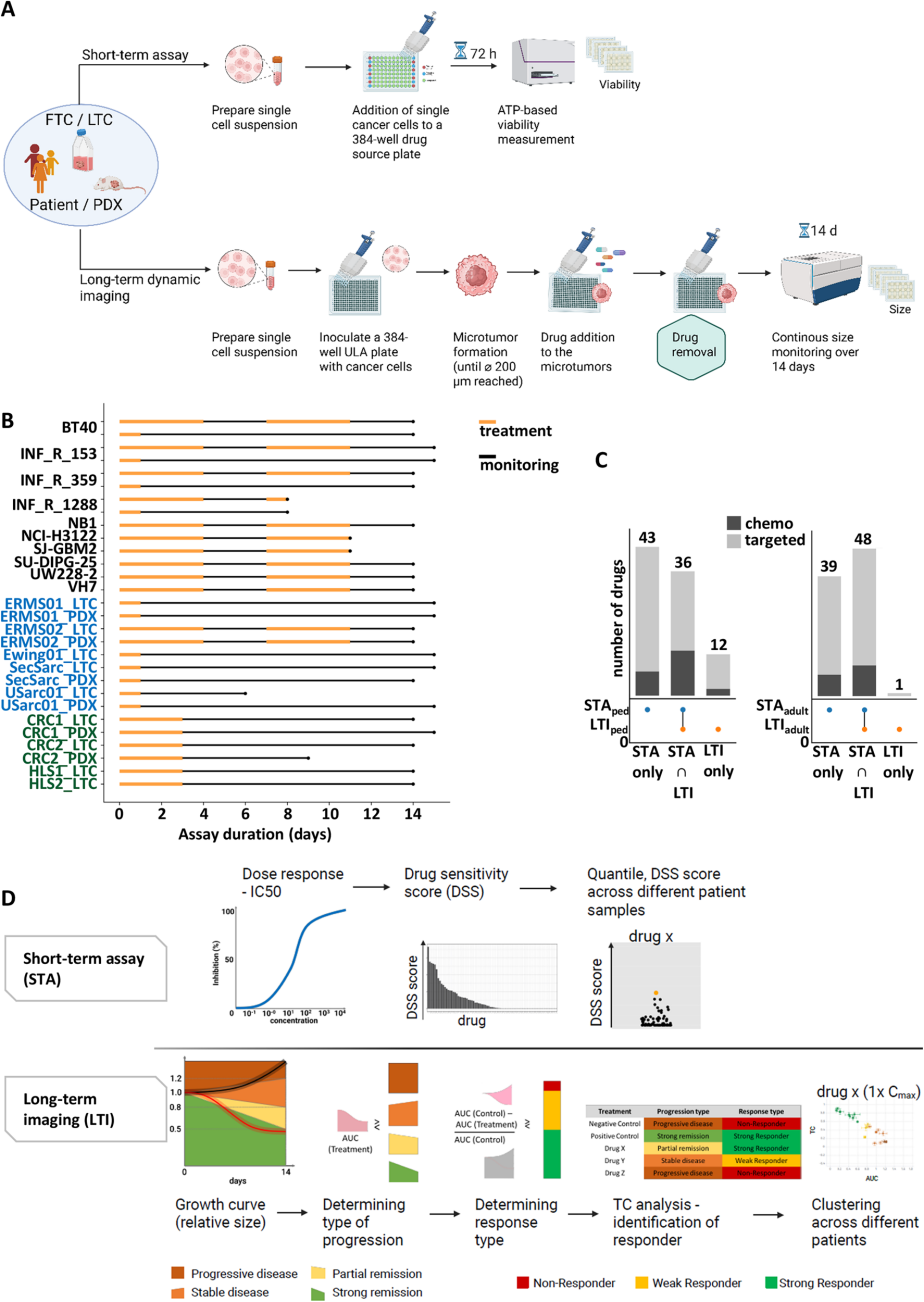
Experimental design. **A** Patient-derived tumor models from long-term cultures (LTC) or patient-derived xenografts (PDX) were subjected to two independent drug sensitivity testing workflows with distinct readouts. For endpoint measurements, an ATP-based viability assay was applied: single-cell suspensions were seeded into 384-well plates containing pre-spotted drug libraries and incubated for 72 h before quantification of cellular ATP levels as a surrogate for viability. Drug sensitivity scores (DSS_asym_) were calculated using standardized statistical models. In parallel, a long-term dynamic assay was used to assess treatment responses via kinetic imaging: after microtumor formation in ultra-low attachment plates, drugs were applied transiently for 24–96 h and removed. Tumor size progression was tracked for up to 16 days by brightfield microscopy. Image segmentation and quantification enabled the calculation of treatment-to-control ratios (TC) and classification of responses according to RECIST-like categories (e.g., stable disease, partial remission, strong remission). The comparison of both approaches aimed to evaluate their concordance and complementary predictive value for functional precision oncology. **B** Segmented lollipop plot illustrating assay duration and treatment exposure for each model. The solid segment represents the treatment phase (24 h–96 h, converted to days), followed by the dashed segment indicating the subsequent drug-free longitudinal monitoring period. The total length of each line corresponds to the overall assay duration, and the black dot marks the endpoint of longitudinal assessment. Models: Black font: positive control models and cell lines; Blue: pediatric patient-derived models; Green: adult patient-derived models. **C** Total number and overlap of drugs screened in each platform. UpSet plots illustrating the intersection of libraries between (left) STA_ped_ and LTI assays and (rightt) STA_adu_ and LTI assays. Upper bar charts indicate the absolute number of drugs exclusively used by one assay (“only”) or shared between assays (“∩”). The lower matrix displays set membership, with filled circles indicating inclusion in a given assay and connecting lines marking intersections. Chemo = chemotherapeutic agents; targeted = molecularly targeted agents. STA_ped_: short-term assay pediatric cancer; STA_adu_: short-term assay adult cancer; LTI: long-term dynamic imaging. **D** Comparative data analysis workflows for endpoint and dynamic drug sensitivity readouts. Top panel: For short-term assays, drug sensitivity was assessed via ATP-based viability measurements after 72 h of continuous drug exposure. Dose–response curves were used to calculate IC_50_ values and drug sensitivity scores (DSS_asym_). These scores were compared with all tested drugs and samples, enabling the stratification of individual drugs and patient models into responders and non-responders based on quantile thresholds. Bottom panel: In the long-term dynamic assay, tumor size progression was monitored by serial imaging over 14 days following a brief drug exposure. Growth curves (relative size over time) were used to determine response types (e.g., progression, stable disease, remission) based on area under the curve (AUC) comparisons between treated and control conditions. Treatment-to-control (TC) ratios enabled classification into response categories aligned with RECIST-like criteria. Final results were clustered across patient models to assess heterogeneity of drug response and to identify clinically relevant responders.

To systematically evaluate drug responses in patient-derived tumor models, we applied two independent drug sensitivity profiling workflows with distinct readouts. Models were established either from long-term cultures (LTC) or directly seeded from dissociated patient-derived xenografts (PDX). In the first approach, treatment effects were assessed using an short-term ATP-based metabolic activity assay (STA) reflecting cell viability. Cell suspensions were seeded into 384-well plates preloaded with drug libraries, exposed continuously for 72 h, and subsequently analyzed for cellular ATP content as a surrogate of viability. Drug sensitivity scores (DSS_asym_) were derived from standardized statistical models^11,16^ and enabled quantitative comparison across drugs and patient samples (Fig. 1A). In the second approach, we implemented a long-term dynamic imaging (LTI) assay designed to capture treatment responses under more physiologically relevant conditions. Following microtumor formation in ultra-low attachment plates, drugs were applied transiently for 24 h–96 h or re-applied after four days off-time (Fig. 1B). Tumor growth trajectories were monitored over 14 days by brightfield imaging, and image segmentation provided quantitative tumor size measurements. The microtumor volume doubling time of the diverse models reflected the diversity of growth kinetics of the different models (Table 1). A broad selection of targeted and chemotherapy drugs were tested across the different drug screening platforms, enabling direct comparison of treatment responses between assays. In total, thirty-six drugs were shared between STA_ped_ and LTI and 48 between STA_adu_ and LTI (Fig. 1C). Based on treatment-to-control (TC) ratios (response type), responses were categorized into non-, weak- and strong-responder and in addition RECIST-like classification criteria were used to characterize the progression type, responses were categorized into progression, stable disease, partial remission, or strong remission (Fig. 1D). Comparative data analysis workflows were established for both assay types. In the short-term setting, dose-response curves from ATP readouts yielded IC_50_ and DSS_asym_ values, allowing for the stratification of patients into responders and non-responders according to DSS_asym_ cohort-based quantile thresholds. In contrast, the dynamic LTI assay relied on longitudinal growth curves and area-under-the-curve (AUC) comparisons between treated and control conditions to determine response and progression categories. Results were clustered across patient-derived models to assess heterogeneity and to highlight clinically relevant drug sensitivities (Fig. 1D). For cross-platform comparison, concordance between assays was defined as the identification of biologically active hits relative to solvent control, whereas qualitative response differences, such as magnitude, durability, and response class were qualitative refinements.

**Table 1:** Characteristics of Patient-Derived Tumor Models and Cell Lines

Indication	Model name	RRID	Genetic alteration	Volume doubling time (days)	Patient characteristics		
					age (y)	gender	treatment status
Ascending colon cancer	CRC1	NA	KRAS p.G12A; PI3K p. H1047R	NA	50	Female	NA
Cecal carcinoma	CRC2	NA	KRAS p.G12C	NA	68	Male	NA
Differentiated liposarcoma	HLS1	NA	NA	NA	73	Male	NA
Diffuse intrinsic pontine glioma	SU-DIPG-25	CVCL_C1N0	H3.3K27M mutation; MYC amplification	2	4	Female	NA
Ductal adenocarcinoma	PC01	NA	KRAS p.Gly12Asp; TP53 p.Val272Met	NA	62	Male	NA
Embryonal rhabdomyosarcoma	ERMS01	NA	FGFR4 mutation	4.5 (LTC), 4 (PDX)	7	Male	Post-therapy
Embryonal rhabdomyosarcoma	ERMS02	NA	MDM2 amplification	NA	17	Female	Post-therapy
Ewing sarcoma	Ewing01	NA	EWSR:FLI1 fusion	3.5 (LTC), 4 (PDX)	6	Female	Post-therapy
Glioblastoma	SJ-GBM2	CVCL_M141	MET amplification	3	6	Female	Post-therapy
Lung adenocarcinoma	NCI-H3122	CVCL_5160	ALK Fusion (EML4-ALK)	4.5	54	Male	NA
Medulloblastoma	UW228-2	CVCL_0572	SHH-activated; TP53 mutation	568^a^	9	Female	NA
Myofibroblastoma	INF_R_153	NA	NTRK fusion	4–5	17	Female	First diagnosis
Myxoid liposarcoma	HLS2	NA	NA	NA	60	Female	NA
Neuroblastoma	NB1	CVCL_1440	ALK amplification	5.5	2	Male	-
Neuroblastoma	INF_R_359	NA	PI3K mutation; MYCN amplification	3–8.5	9	Male	Post-therapy
Pilocytic Astrocytoma	BT40	NA	BRAF V600E mutation	4.5	14	Male	-
Rhabdoid tumor	INF_R_1288	NA	SMARCB1 deletion	1	4	Male	Post-therapy
Secondary sarcoma (RT-induced)	SecSarc01	NA	MET mutation; NF1 mutation	7.5 (LTC), 99.5 (PDX)	18	Male	Post-therapy
Undifferentiated sarcoma	USarc01	NA	EML4:ALK fusion	2 (LTC), 131 (PDX)	16	Female	Post-therapy

*NA* not applicable.

^a^ stable in size.

### Cross-validation of assay platforms using models with defined oncogenic drivers

**Fig. 2:**
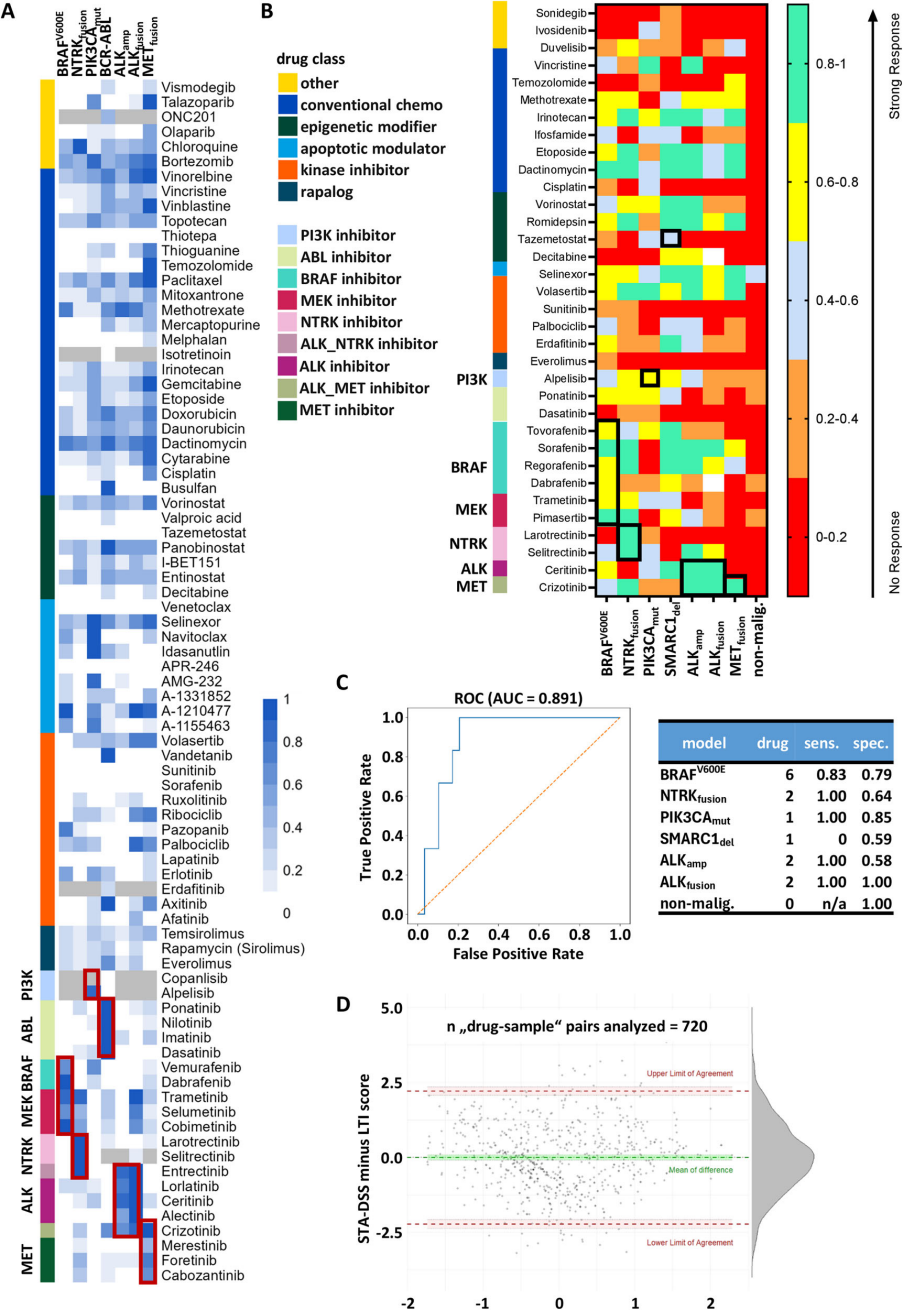
Assay validation using positive control models with known molecular drivers. Heatmaps showing drug response profiles for a panel of positive control models, i.e. established reference cell lines harboring known oncogenic drivers (e.g., BRAF^V600E^, NTRK _fusion_, PIK3CA_mut_, ALK_amp/fusion_, MET_fusion_; model details are listed in Table 1). These models were screened across a broad panel of drugs using short- and long-term dynamic assay formats. **A** ATP-based STA after 72 h of continuous drug exposure. Drugs are grouped by molecular target (e.g., ALK, MEK, BRAF; drug class legend is shared with panel B), and models were evaluated for expected target-matched inhibitor responses. Red boxes indicate exemplary model-drug matches where known target dependencies were captured. **B** Dynamic LTI assay summarizing drug responses of reference models with defined molecular alterations. Drugs are grouped by molecular target. Heatmap colors indicate the normalized response score, ranging from strong response (green) to no response (red), as shown on the color scale. Examples highlighted in black boxes indicate model-drug pairs with known target dependencies. **C** Left panel: Receiver operating characteristic (ROC) curve showing the performance of the long-term imaging (LTI) score to discriminate genotype-matched (positive class) from non-matched drug–model pairs in the BRAF^V600E^-driven BT40 model. The area under the curve (AUC) is indicated. The cutoff LTI ≥ 0.7 was applied as a threshold for all other models for comparison sensitivity/specificity analyses (Table right panel). **D** Bland-Altman analysis visualizing the agreement between the ATP-based endpoint assay and the long-term imaging-based assay across all tested drug-model pairs (*n* = 720). The x-axis shows the mean of the drug sensitivity scores (DSS_asym_) derived from both assays, while the y-axis displays the difference between ATP-based and imaging-derived scores for each treatment condition. The green dashed line represents the mean difference, while the red dashed lines denote the upper and lower limits of agreement (mean ± 1.96 × standard deviation). A density plot on the right illustrates the distribution of differences. The majority of points fall within the agreement interval, indicating a good overall concordance between the two assay platforms despite methodological differences in readout and kinetics.

To cross-validate both assay platforms (STA and LTI), we tested whether each platform could detect biologically expected drug sensitivities based on known genotype-drug target relationships across different pediatric cancer types. Specifically, we hypothesized that models harboring defined oncogenic driver alterations would display selective sensitivity to their corresponding targeted inhibitors across both assay formats. To address this, we first applied them to a panel of positive control models with well-characterized oncogenic drivers, including BRAF^V600E^ ^17^, NTRK fusion (Supplementary Fig. 1A), PIK3CA mutation^18^, ALK amplification and fusion^19,20^, as well as MET fusion^21^. These models were screened against the overlapping set of drugs that included amongst others targeted agents matched to the underlying oncogenic drivers. In the short-term ATP-based assay (STA), cells were exposed to drugs continuously for 72 h, whereas in the long-term dynamic imaging (LTI) assay, treatment was applied transiently and followed by longitudinal imaging of tumor growth over approx. 14 days. As expected, both readouts captured matched drug-target dependencies associated with the underlying molecular alterations. Figure 2A shows the STA results, whereas Fig. 2B shows the corresponding LTI assay. The heatmaps highlight exemplary model-drug pairs. Together, these results demonstrate that dynamic LTI size-based profiling recapitulates the expected response patterns, also observed in ATP-based STA in reference models with defined molecular drivers (Fig. 2A,B). To further evaluate the LTI assay performance to discriminate genotype-matched (positive class) from non-matched drug–model pairs in the BRAF^V600E^-driven BT40 model, we performed a Receiver operating characteristic (ROC) curve analysis (Fig. 2C).

To quantify assay performance across models, sensitivity and specificity were calculated on a model-specific level using a predefined response threshold (LTI score ≥ 0.7). Drug-model pairs were classified as positive or negative based on this cutoff and compared to the expected genotype-drug matches. In models with at least one matching drug, the mean sensitivity was 81%, while the mean specificity across all models was 78% (Fig. 2C, Supplementary Data 1).

To formally evaluate concordance between the two assay types, we performed a Bland-Altman analysis across all drug-model pairs with complete data in both assays. The mean sensitivity scores from both assays (DSS_asym_ for the ATP-based STA and normalized AUC for the LTI assay) were compared, and the differences were plotted against their overall average response. A total of 96.5% of the observations fell within the 95% limits of agreement. These findings indicate a strong concordance between ATP-based STA and LTI-based profiling despite differences in assay kinetics and readout (Fig. 2D).

### Short- and long-term functional drug testing provide complementary insights into drug response in pediatric and adult cancers

**Fig. 3:**
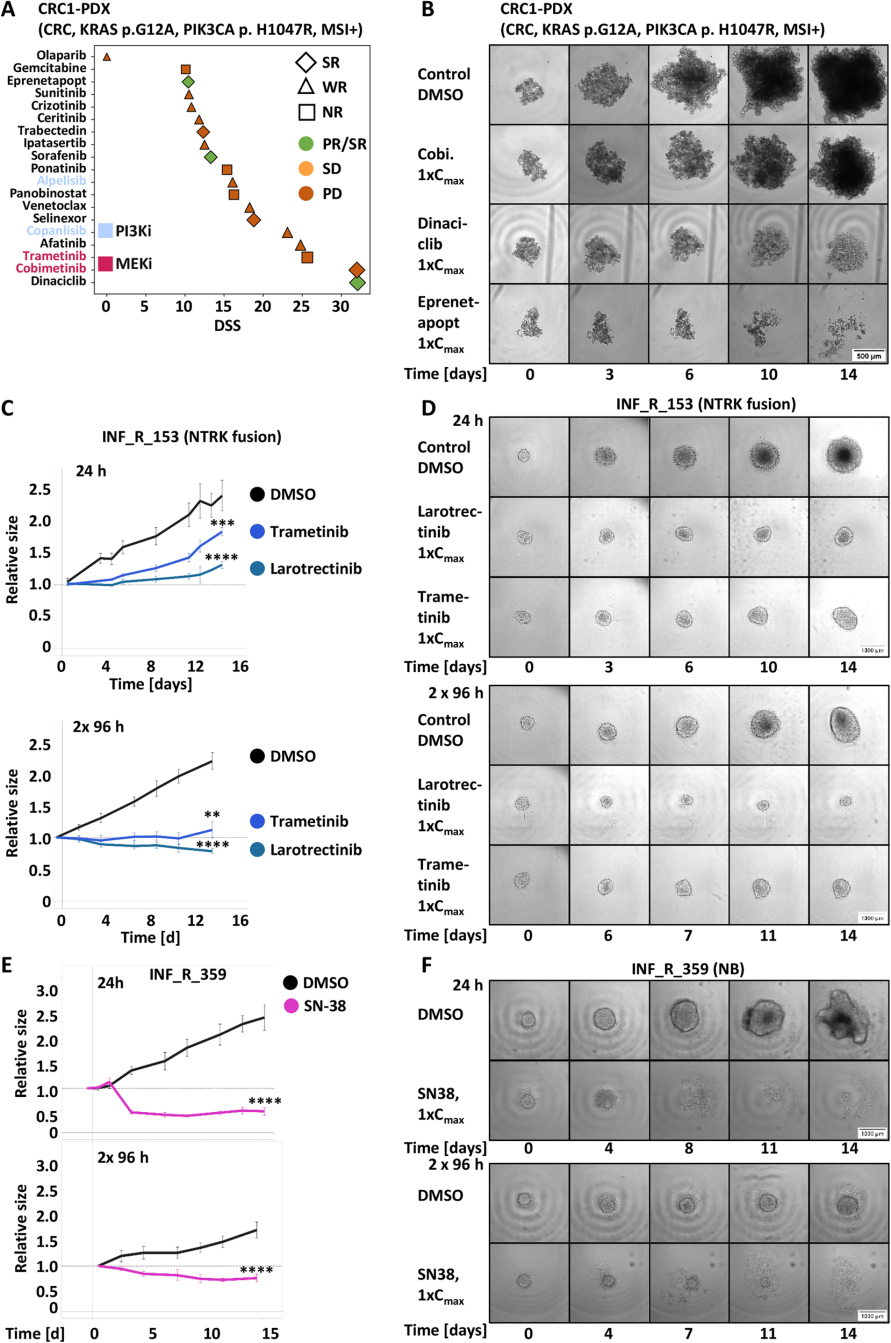
Dynamic imaging of gene alteration-driven models. **A** Comparative analysis of STA and LTI assays with the genomic complex adult colon cancer CRC1 model harboring KRAS and PIK3CA mutations (details see Table 1 and Supplementary Table 3). The x-axis represents DSS values measured by STA, while each row corresponds to an individual drug. Marker shape indicates response type (SR: strong responder, WR: weak responder, NR: non-responder), and marker color reflects imaging-based RECIST-like progression categories (SR: strong remission, SD: stable disease, PD: progressive disease). Marker size is scaled proportionally to DSS. **B** Corresponding images to the model described in (**A**). Depicted are the 1xC_max_ treatments with cobimetinib (Cobi), dinaciclib, eprenetapopt and the corresponding solvent control (DMSO). Microtumors were imaged for 14 days. The dynamic imaging reveals predominantly cytostatic rather than cytotoxic effects upon cobimetinib treatment. Cell debris is visible in addition to viable cells for dinaciclib and eprenetapopt at later timepoints. Scale bar 500 µm. **C** Microtumor growth curves for two different treatment regimens of the NTRK_fusion_ model INF_R_153 (model details in Table1). The upper graph displays the relative growth (change from baseline) after one-time treatment with MEKi trametinib (*n* = 3), NTRKi larotrectinib (*n* = 3), or DMSO solvent control (*n* = 26), respectively, for 24 h. The lower graph depicts growth kinetics for trametinib (*n* = 3), larotrectinib (*n* = 3), or DMSO (*n* = 23) from the long-term imaging assay under a 96 h ON / 3 days OFF / 96 h ON treatment schedule. The relative growth shows the fold-increase in relative size (diameter) over 14days. Error bars represent SD. Statistics: Last time point comparison to DMSO. One-way ANOVA followed by Dunnett’s multiple comparisons test. ** *p* = 0.0011; *** *p* = 0.0006, **** *p* < 0.0001. **D** Corresponding images to (**C**). Microtumors were treated with 1xC_max_ concentrations for the indicated times and imaged for 14 days. Scale bar 1000 µm. **E** Microtumor growth curves for chemotherapy (SN-38) treatment of the neuroblastoma model INF_R_359 (model details in Table1). The upper graph displays the relative growth (change from baseline) after one-time treatment with the active metabolite of irinotecan, SN-38 (*n* = 3), respectively, for 24 h and DMSO control (*n* = 13). The lower graph depicts growth kinetics for SN-38 (*n* = 3) and DMSO (*n* = 13) from the long-term imaging assay under a 96 h ON / 3 days OFF / 96 h ON treatment schedule. The relative growth shows the fold-increase in relative size (diameter) over 14 days. Error bars represent SD. Statistics: Unpaired t- test. **** *p* < 0.0001. **F** Corresponding images to (**E**). Microtumors were treated with 1xC_max_ concentrations for the indicated times and imaged for 14 days. Scale bar 1000 µm.

Although both platforms detect pharmacologically active drugs, the long-term imaging assay captures response dynamics that are not accessible through short-term endpoint measurements. After establishing cross-platform concordance in pediatric reference models, we applied both assays to additional pediatric and adult patient-derived cancer models. This broader comparison further illustrates how longitudinal response profiling complements short-term drug sensitivity testing across diverse tumor contexts (Fig. 3A) in a clinically relevant setting with high biological complexity (PDX-derived model, details Supplementary Table 3). Drugs were tested at clinically achievable concentrations, ensuring that observed responses reflect pharmacologically relevant exposure ranges. Both assays generated drug sensitivity profiles that could be directly compared across platforms. DSS yielded a ranked list of drug activities. While effect magnitude and response quality differ, the majority of active drugs are detected as hits by both assays (Fig. 3A). Highest-scoring drugs in the STA were MEK inhibitors (cobimetinib, trametinib), PI3K inhibitors (copanlisib), as well as CDK inhibitor dinaciclib. LTI provided additional information on treatment durability, including regrowth after drug wash-out (MEKi) and differentiation between cytostatic (weak or strong response, but no shrinkage of microtumor size) and cytotoxic (regression; decrease in microtumor size) effects (Fig. 3A,B**;** Supplementary Fig. 3A,B). For example, MEKi such as cobimetinib slowed down proliferation compared to DMSO-treated microtumors without regression, which is consistent with cytostatic activity. In contrast, dinaciclib induced strong response (SR), which is indicative of cytotoxic effects (Supplementary Fig. 3A). Differences in response quality and classification therefore likely reflect the distinct biological questions addressed by the two assay formats. These findings illustrate that functional testing can reveal pharmacologically exploitable vulnerabilities and identify candidate therapies even in genomically complex models.

Furthermore, to simulate pharmacokinetics, we selected short-half-life targeted drugs according to the clinically inspired intermittent treatment protocols. This involved drug exposure for four days (96 h), medium change and a three-day incubation period without the drug, followed by re-drugging for another four days (Figs. 3C-E, 1B). Compared to the one-time, short treatment (24 h), the positive control INF_R_153 model with the NTRK fusion showed the expected strong response after the two 96 h regimen simulations. The NTRKi larotrectinib resulted in more sustained growth inhibition than other targeted drugs (e.g., the MEKi trametinib) across both treatment schedules (Fig. 3C, D). In line with their pharmacokinetic properties, the standard-of-care chemotherapeutics irinotecan (active metabolite SN-38, Fig. 3E) and dactinomycin (Supplementary Fig. 3B,C) were similarly effective in the INF_R_359 neuroblastoma model or the differentiated liposarcoma HLS1 model, respectively, under the 24 h protocol and the longer treatment conditions.

### Dynamic long-term imaging extends drug response interpretation beyond short-term ATP-based assays

In addition to validating the assay on reference models, we systematically compared its performance across a broader set of models, including patient-derived microtumors (Table 1, Fig. 1B). Notably, dynamic LTI confirmed 29 out of 40 drug hits (73%) identified in the STA (Supplementary Fig. 3, Supplementary Table 2). Furthermore, imaging provided a more detailed qualitative characterization of the drug effects and identified ex vivo stable disease (SD) and disease that was still progressive (PD) but less proliferative than the control (Supplementary Fig. 3, Supplementary Table 2). These data underscore the value of the LTI platform in distinguishing ex vivo growth inhibition (cytostatic) from tumor regression (cytotoxicity). This allows for a more nuanced assessment and refines the interpretation of drug responses detected in ATP-based endpoint measurements, providing a more accurate evaluation of therapeutic activity than end-point-only ATP measurement.

### Dynamic LTI assays extend endpoint testing by capturing treatment kinetics of drug combinations

Having established the complementary insights provided by STA and LTI assays, we applied the long-term dynamic imaging to evaluate drug combination effects in patient-derived tumor models. Combination therapies are the clinical standard for many cancers and are often required to overcome resistance to single-agent therapies. However, functional testing of combination treatments remains challenging because potential interaction effects and dosing constraints can influence response interpretation. To address this, we included a panel of combinations comprising clinically established regimens, such as dabrafenib/trametinib, as well as exploratory combinations selected based on complementary mechanisms of action and preclinical evidence of enhanced antitumor activity (Supplementary Table 4).

**Fig. 4:**
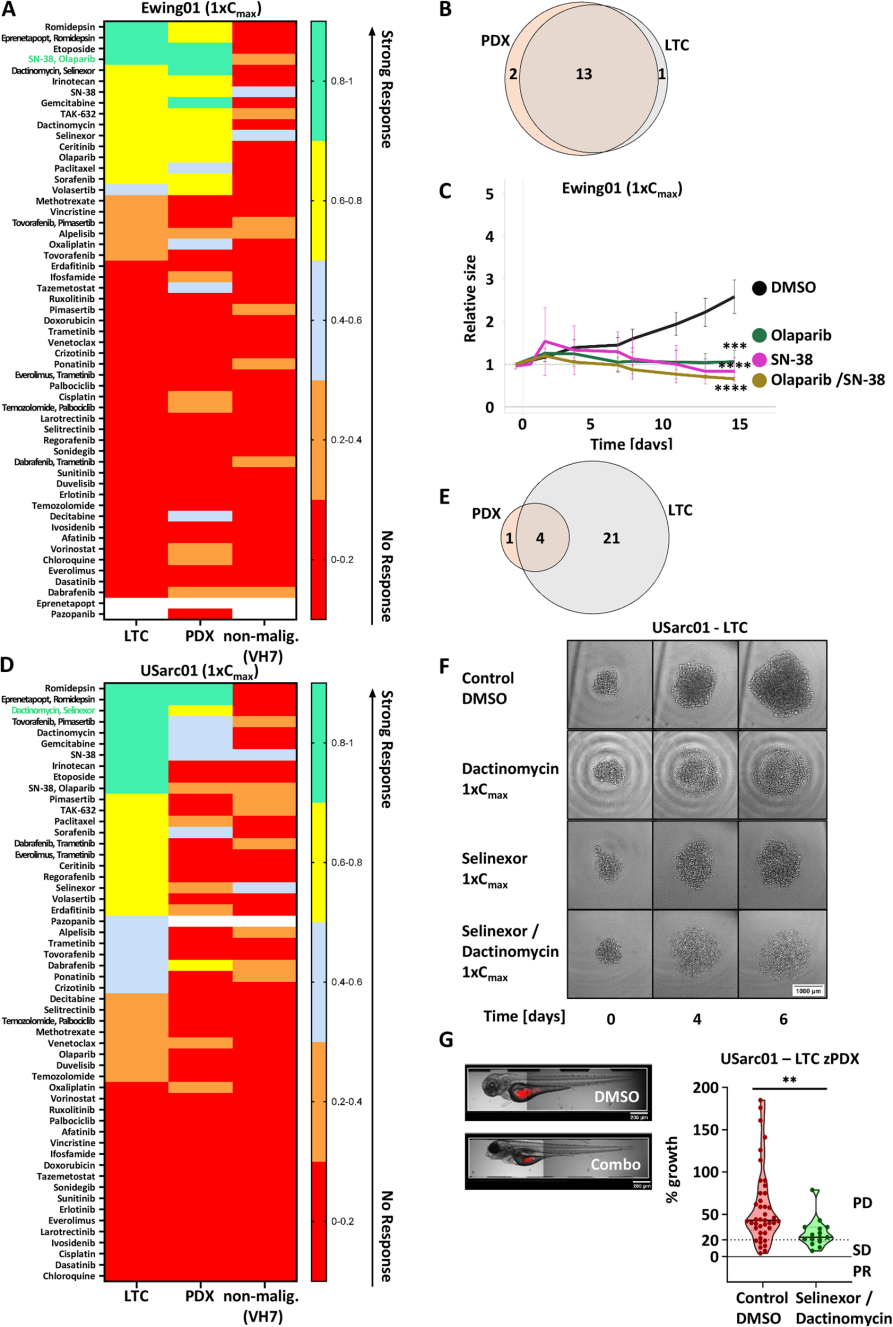
Drug responses in the pediatric patient-derived models across PDX and LTC platforms. **A** Heatmap summarizing the drug responses observed in the patient-derived Ewing01 model using long-term cultures (LTC) or PDX-derived cells (PDX) in comparison to non-malignant control cells (VH7). Model details are listed in Table 1 and Supplementary Table 3. For each drug, response type at 1×C_max_ is classified in five categories from strong responders (green) to no response (red). **B** Venn diagram illustrates the overlap in drug hits between both Ewing01 model systems. Of 16 total strong responses, 13 were shared, 2 were unique to PDX, and 1 to LTC. **C** Line plot shows relative growth (change from baseline) from dynamic LTI after one-time treatment (see Fig. 1B) with a selected combination treatment (Olaparib + SN-38; *n* = 3) and monotherapies (each *n* = 3), compared to DMSO control (*n* = 11). The combination induced sustained growth suppression over 14 days. The relative growth shows the fold-increase in relative size (diameter) over 14days. Error bars represent SD. Statistics: Last time point comparison to DMSO. One-way ANOVA followed by Dunnett’s multiple comparisons test. *** *p* = 0.0001, **** *p* < 0.0001. **D** Heatmap summarizing the drug responses observed in the patient-derived USarc01 model using long-term cultures (LTC) or PDX-derived cells (PDX) in comparison to non-malignant control cells (VH7). For each drug, response type at 1×C_max_ is classified in five categories from strong responder (green) to no response (red). **E** Venn diagram illustrates the overlap in drug hits between both USarc01 model systems. Of 26 total strong responses, 4 were shared, 1 was unique to PDX, and 21 to LTC. **F** Brightfield images showing the growth of USarc01-LTC microtumors over 6 days in vitro following treatment with dactinomycin, selinexor, or the combination at 1×Cmax, compared to DMSO control. While both monotherapies induced partial growth inhibition, the combination resulted in marked growth arrest or regression. **G** Left: Representative fluorescent images of zebrafish embryos xenografted with DiI-labeled USarc01-LTC cells and treated for 48 h with the combination of selinexor and dactinomycin. Treated embryos display reduced tumor burden compared to controls. Right: Violin plot showing pooled quantitative analysis of tumor growth in zebrafish embryos at 48 h post-treatment. The combination significantly reduced tumor growth compared to DMSO (Mann–Whitney test, *p* = 0.0023), confirming the in vitro findings in an in vivo setting.

Patient-derived cancer models were exposed to these combinations at three concentrations (1×C_max_, 0.1×C_max_, and 0.01×C_max_), spanning clinically relevant to sub-therapeutic levels. Treatment responses were monitored by LTI over approximately 2 weeks, allowing the assessment of ex vivo long-term treatment kinetics and durability of response. For example, we applied dynamic imaging on a patient-derived Ewing sarcoma model harboring an EWSR:FLI1 fusion. The dynamic screen yielded 14 strong drug responses, including two combination treatments, classified as strong responders according to the obtained TC ratio (Fig. 4A**;** Supplementary Fig. 5A,B). The assay duration was 16 days and drugs were applied for the first 24 h after spheroid formation (initial diameter ~200 µm).

To test assay performance using freshly derived tumors, corresponding tumor tissue from the same patient was xenotransplanted into NSG mice (NOD.Cg-Prkdc^scid^ Il2rg^tm1Wj^l/SzJ). When resulting PDX tumors reached required size, we explanted fresh tissue, dissociated the cells and seeded them as a fresh-tissue microtumor culture (PDX_FTC), before applying dynamic LTI. The PDX-derived FTC yielded 15 strong responder hits (Fig. 4A, Supplementary Fig. 2A,B), with an overlap of 13 drugs compared to the LTC screen (Fig. 4B). Among the combination treatments, SN-38 (the active metabolite of the chemotherapeutic drug irinotecan) together with a PARP inhibitor (olaparib) showed particular strong activity (Fig. 4C, highlighted in green). This combination has been proposed before for the treatment of children and young adults with recurrent or refractory solid malignancies, especially Ewing sarcomas^22,23^ and is currently tested in clinical trials (ClinicalTrials.gov NCT02044120, 2014-01-21; NCT02813135, 2016-06-16; Supplementary Table 4). Consistent with these reports, the dynamic imaging assay captured marked treatment responses in the Ewing sarcoma model, supporting the potential utility of the assay for ex vivo assessment of combination efficacy in defined molecular contexts.

In a similar experiment we applied dynamic LTI to patient-derived undifferentiated sarcoma model harboring an EML4:ALK fusion (USarc01). The assay identified 25 strong responder hits (Fig. 4D, Supplementary Fig. 1B,C, Supplementary Fig. 4A). The total assay duration was nine days, corresponding to the time point when the spheroids reached their maximal size. Drugs were applied for the first 24 h after spheroid formation (with an initial diameter of 200 µm). For this case the corresponding PDX model yielded 5 strong responder hits, of whose 4 overlapped with the LTC-LTI screen (Fig. 4E). Among the combination treatments tested, the combination of dactinomycin and selinexor induced pronounced growth inhibition (Fig. 4D,F). Brightfield imaging over six days showed that both monotherapies induced partial tumor growth inhibition. However, their combination at 1× C_max_ resulted in pronounced and sustained growth arrest and regression compared to controls treated with DMSO (Fig. 4F). Zebrafish embryo xenografts were used as a rapid in vivo-proximal model to assess whether drug-induced effects observed in ex vivo assays translate into altered tumor growth behavior within a living organism. To further validate the ex vivo findings, we tested the selinexor/dactinomycin combination in a zebrafish patient-derived xenograft (zPDX) model transplanted with USarc01-LTC cells. Consistent with the dynamic imaging findings, fluorescent imaging of zebrafish embryos xenografted with CM-DiI -labeled USarc01-LTC cells revealed significantly (Mann–Whitney test, *p* = 0.0023) reduced tumor burden after 48 h of combination treatment (*n* = 15) compared to the solvent group (*n* = 41) (Fig. 4G). While median relative tumor growth in the DMSO group was 43% (maximum tumor growth 185%, minimum tumor growth 4%), median tumor growth decreased to 23% in the selinexor/dactinomycin group (maximum tumor growth 79%, minimum tumor growth 7%) despite the short treatment time of 48 h. The same combination treatment was tested in zPDX using the Ewing01 model, which showed a weaker response in the LTI. Here, the zPDX showed a similarly weaker response, with a reduction in progressive tumors (PD) from 80% to 44% (median growth from 30% DMSO to 20% combo) demonstrating concordant treatment responses between the ex vivo imaging assay and the in vivo zPDX model (Supplementary Fig. 4B). A systematic comparison of drug response profiles between mouse(m)-PDX derived short-term screens and the corresponding long-term culture (LTC) model screens revealed an overall good correlation of drug response between FTC-mPDX- and LTC-mPDX (Supplementary Fig. 6). However, certain drugs were preferentially active in one model system, such as ceritinib, erdafitinib, crizotinib, and trabectedin in PDX-derived cultures, whereas SN-38, volasertib, and paclitaxel showed stronger activity in long-term culture models.

**Fig. 5:**
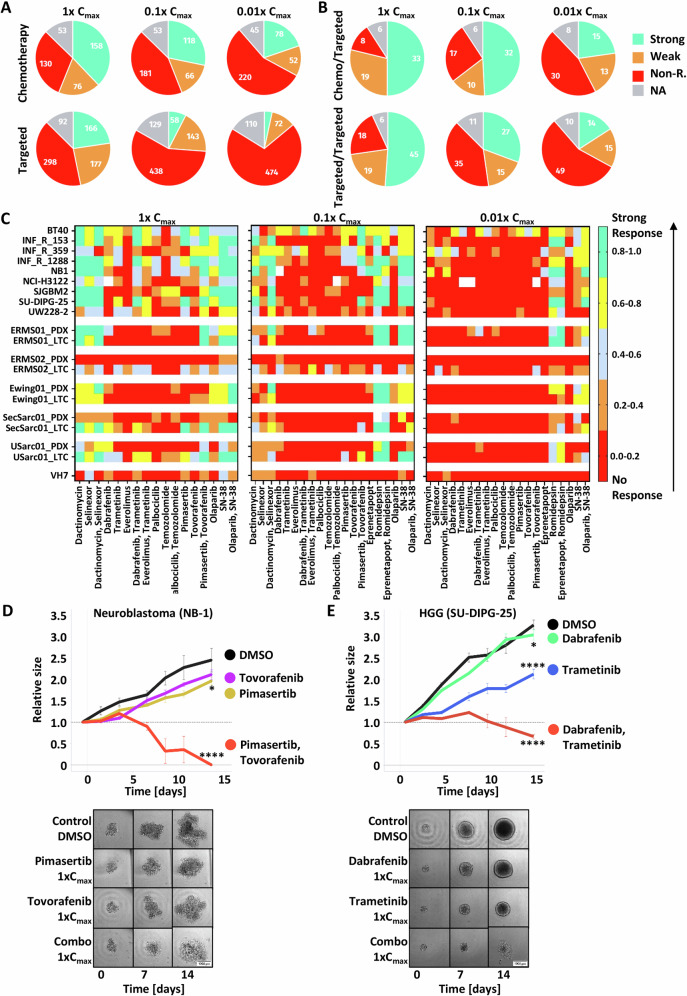
Beneficial effects of combination treatments. **A**, **B** Response classification across drug classes and concentrations in dynamic imaging-based assays. Pie charts illustrate the distribution of treatment responses across all tested patient-derived cancer models (see Table 1 and Supplementary Table 3) for three drug categories: **A** chemotherapy and targeted agents, and **B** drug combinations. Two types of combinations were tested: chemotherapy/targeted agent pairs (top row) and targeted/targeted agent pairs (bottom row). Drugs were tested at three concentrations corresponding to 1×, 0.1×, and 0.01×C_max_. Responses were classified into strong responders (green), weak responders (orange), non-responders (red), and not available (NA) (gray), based on tumor size progression following short-term drug exposure (24–96 h) and 14-day imaging-based monitoring. Across all concentrations, combination therapies yielded a higher proportion of responding models compared to monotherapies, with the effect most pronounced at 1×Cmax. **C** Heatmaps summarize treatment responses of patient-derived cancer models to a broad panel of drug combinations at three concentrations: 1×C_max_ (left), 0.1×C_max_ (center), and 0.01×C_max_ (right). Each row represents a patient-derived model (PDX or LTC), and each column corresponds to a specific drug combination. Colors indicate normalized treatment response scores, ranging from strong response (green) to no response (red), based on tumor size progression over 14 days after short-term drug exposure. The tested combinations include both chemotherapy/targeted and targeted/targeted agent pairs. Some combinations (e.g. roniciclib/eprenetapopt) could not be tested at 1×C_max_ due to solubility constraints. The data illustrate heterogeneous and concentration-dependent response patterns across models and combinations. **D**, **E** Line plots displaying representative growth curves from dynamic imaging for selected combination treatment (**D**: DMSO (*n* = 10), tovorafenib (n = 3), pimasertib (*n* = 2), tovorafenib + pimasertib (*n* = 3); **E** DMSO (*n* = 13), trametinib (*n* = 3), dabrafenib (*n* = 3), dabrafenib + trametinib (*n* = 2)). In contrast to the monotherapy, the combination induced growth remission over 14 days, suggesting functional synergy in these models. The relative growth shows the fold-increase in relative size (diameter) over 14days. Error bars represent SD. Statistics: Last time point comparison to DMSO. One-way ANOVA followed by Dunnett’s multiple comparisons test. * *p* < 0.05, **** *p* < 0.0001. Lower left panels (**D**): Brightfield images showing the growth of neuroblastoma NB-1 microtumors over 14 days in vitro following treatment with tovorafenib, pimaserib, or the combination at 1×C_max_, compared to DMSO control. Lower right panels (**E**): Brightfield images showing the growth of high-grade glioma (HGG) SU-DIPG-25 microtumors over 14 days in vitro following treatment with dabrafenib, trametinib, or the combination at 1×C_max_, compared to DMSO control. Scale bar: 1000 µm.

A comprehensive analysis of all screens compared combination treatments with single-agent targeted drugs or chemotherapeutic drugs. This analysis revealed higher proportions of strong responders compared to single-agent assays, increasing from 23% (166/733 targeted) or 38% (158/417 chemotherapy) to 50% (33/66) for “chemotherapy-plus-targeted” combinations and 51% (45/88) for “targeted-plus-targeted” combinations (Fig. 5A, B). Heatmaps summarizing the data of *n* = 20 distinct models revealed pronounced heterogeneity across models, with diverse sensitivities observed in FTC-mPDX and LTC. Response strength generally decreased in a concentration-dependent manner, but specific tested combinations maintained activity even at lower concentrations (Fig. 5C). Notably, some combinations, such as romidepsin/eprenetapopt, could not be evaluated at 1×C_max_ due to solubility limitations. Furthermore, certain combinations shifted treatment responses from cytostatic to cytotoxic. For example, MEKi plus RAFi combinations can do so in the NB-1 or SU-DIPG-25 model (Fig. 5D,E). These findings highlight the heterogeneous and concentration-dependent nature of combination treatment responses across patient-derived models. Collectively, dynamic LTI assays capture heterogeneous and concentration-dependent responses to drug combinations and thereby can complement monotherapy testing.

### Clinical case study illustrating the translational relevance of longitudinal response profiling

**Fig. 6:**
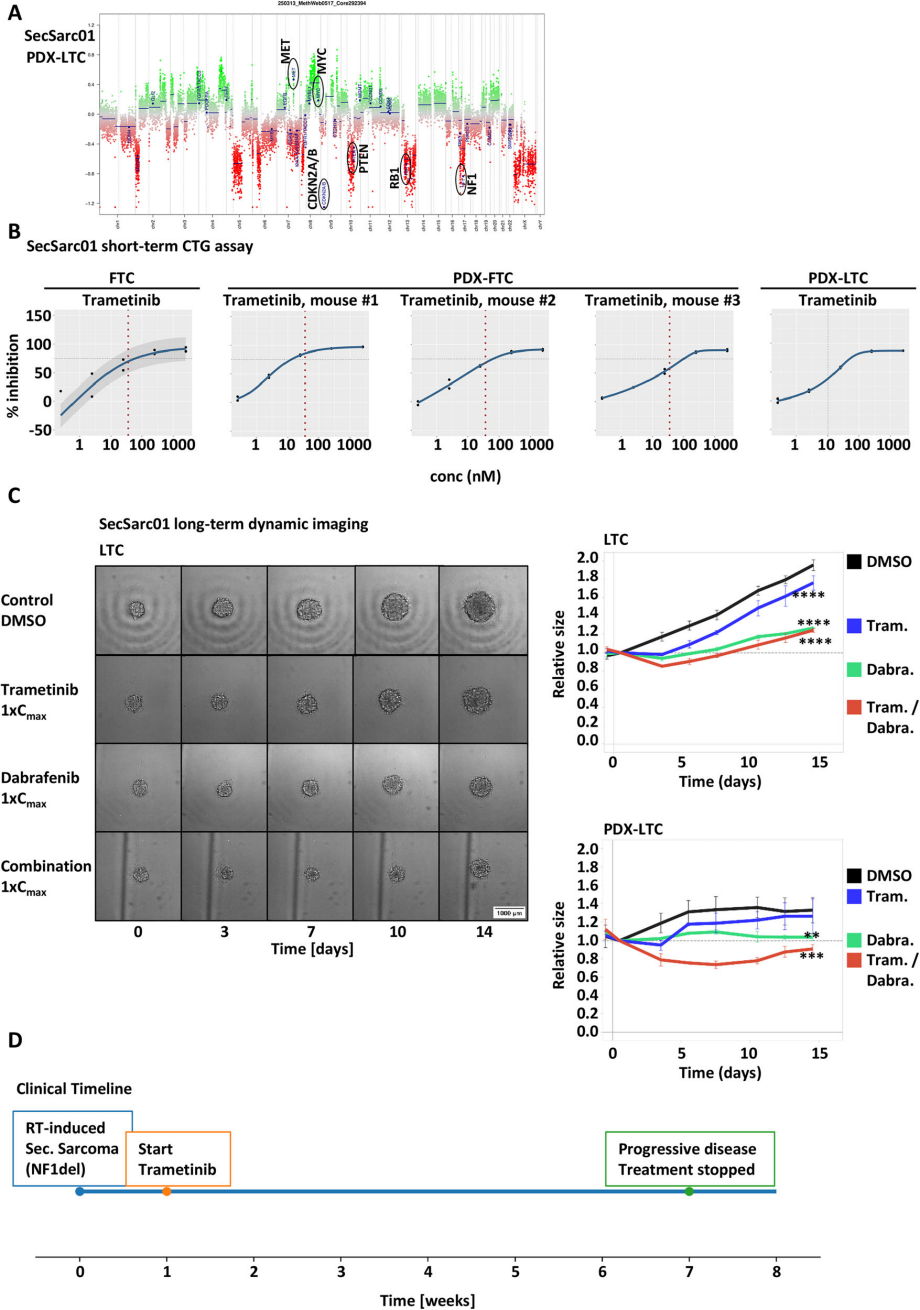
Clinically annotated proof-of-principle case illustrating assay-specific response interpretation. **A** Copy number aberration (CNA) plot of the radiotherapy-induced secondary sarcoma showing key alterations, including NF1 deletion. **B** Short-term ATP-based assay (STA) identifies MEK inhibition (trametinib) as a potential hit in fresh tissue culture (FTC), PDX-FTC and PDX-long-term culture (LTC) screens. Model details are listed in Table 1 and Supplementary Table 3. **C** Long-term imaging (left side; scale bar, 1000 µm) and LTI-based growth curves (right side) demonstrate only transient growth suppression followed by regrowth after trametinib withdrawal. The relative growth shows the fold-increase in relative size (diameter) over 14days. DMSO *n* = 13 each model; dabrafenib *n* = 3 each; trametinib, *n* = 3 each; trametinib + dabrafenib *n* = 3 SecSarc01_LTC and *n* = 2 SecSarc01_PDX-LTC. Error bars represent SD. Statistics: Last time point comparison to DMSO. One-way ANOVA followed by Dunnett’s multiple comparisons test. ** *p* = 0.0026, *** *p* = 0.0002, **** *p* < 0.0001. **D** Clinical timeline of the patient with NF1-deleted secondary sarcoma, indicating initiation of trametinib therapy and subsequent discontinuation due to progressive disease.

To illustrate the translational implications of assay-specific response profiles, we analyzed a clinically annotated case of a radiotherapy-induced secondary sarcoma harboring an NF1 deletion (Fig. 6A). The ATP-based STA identified MEK inhibition (trametinib) as a potential hit in patient-derived FTC, PDX-derived FTC, and PDX-derived LTC (Fig. 6B). However, the LTI assay revealed only weak, nondurable growth suppression (Fig. 6C). The patient received trametinib, but developed progressive disease after six weeks of treatment, consistent with the LTI findings (Fig. 6D).

## Discussion

The landscape of functional precision oncology is rapidly evolving, driven by the need to complement histopathological and molecular diagnostics with assays that directly measure tumor-specific drug responses. While next-generation sequencing (NGS) has become a cornerstone of precision oncology^15,24–28^, its predictive power remains limited in many patients, as not all genomic alterations translate into druggable dependencies^1,29^. Functional assays provide an orthogonal readout that can uncover vulnerabilities beyond genomics^1^ and are increasingly explored as a decision-support tool in precision oncology, yet its translational interpretation critically depends on assay design and readout. Systematic benchmarking of conceptually distinct platforms is therefore essential to understand their respective strengths and limitations and to guide their optimal use in clinical decision-making.

Several functional assay formats based on different patient-derived tumor models have been used to identify drug responses ex vivo. These range from simple viability assays using dissociated cells to growth dynamics in complex patient-derived organoid (co-) cultures and patient-derived xenografts (PDX), which together offer unique and complementary insights into tumor biology and therapeutic vulnerabilities^6,30–33^.

Furthermore, PDX-derived cells and organoids, including those generated by enzymatic dissociation, have been widely established as functional precision oncology platforms that preserve key tumor-specific characteristics and enable clinically relevant drug response profiling across cancer types^34–42^. However, despite the growing number of functional precision oncology studies, systematic comparisons between commonly used assay formats applied to identical patient-derived models remain scarce. This assay diversity also highlights a critical challenge because no single approach can fully capture the multifaceted nature of tumor-drug interactions across different clinical and developmental contexts. Instead, an integrated approach that combines the strengths of multiple assays may provide the most robust framework for both individualized patient care and rational drug development. Such integration requires systematic validation, cross-platform benchmarking, and a clear understanding of how each assay’s design, readout, and biological complexity influence its predictive power. As functional assays gain traction in clinical trials and early drug discovery pipelines, defining their optimal roles and inter-dependencies will be essential to realize the full potential of functional precision oncology.

Therefore, our study directly addresses one of the pressing challenges in functional precision oncology, the lack of systematic cross-platform benchmarking. By comparing two conceptually distinct approaches, a short-term ATP-based viability assay and a dynamic long-term imaging assay across a broad spectrum of patient-derived cancer models, we observed robust cross-platform consistency and consistently identified responders and non-responders in alignment with molecular expectations. Strong concordance between independent functional testing platforms supports the biological robustness and translational reliability of functional drug response profiling, thereby strengthening confidence in its clinical utility. While the ATP-based assay excels in speed and scalability, providing a rapid assessment of pharmacologically active drugs within approximately three days, the imaging-based platform captures treatment dynamics over an extended observation period of up to two to three weeks. This longitudinal monitoring distinguishes transient growth suppression from durable stabilization or tumor regression, response patterns that more closely resemble clinically relevant treatment outcomes^34,43,44^. These complementary characteristics suggest distinct clinical use cases: rapid short-term assays may support treatment prioritization in patients requiring urgent therapeutic decisions, whereas longitudinal profiling may be particularly valuable for evaluating off-label therapies or drug combinations where understanding response durability and interaction effects is critical.

Our cross-platform validation shows that cohort-based ATP-based viability profiling and dynamic imaging provide highly concordant yet complementary results, capturing distinct aspects of tumor response. Although conceptually different, both assays identified drug-target dependencies consistent with molecular alterations, supporting previous observations on the utility of functional assays in precision oncology^45^. These results extend previous work by directly comparing two distinct platforms within the same patient-derived models, showing that rapid viability assays efficiently detect active drugs, whereas imaging adds interpretative depth by distinguishing cytostatic from cytotoxic effects. A similar observation was made in epithelial cancer organoids, where dynamic profiling was required to uncover treatment effects that short-term viability failed to detect^46,47^. Furthermore, the ATP-based approach relies on comparison with a reference cohort, and its interpretive power increases with cohort size. In contrast, the dynamic long-term imaging assay evaluates treatment responses longitudinally within individual samples and can therefore generate informative readouts even in the absence of large comparative datasets. This feature may be particularly valuable in rare cancers or highly individualized clinical settings.

Our comparative analysis illustrates that short-term metabolic viability assays (STA) and long-term imaging-based growth (LTI) assays capture distinct biological response dimensions and qualities, reflecting acute drug-induced metabolic effects versus the durability of growth suppression after transient exposure. These assay-intrinsic differences may be particularly relevant for drugs with predominantly cytostatic modes of action and highlight the importance of considering assay-specific response dynamics when interpreting functional drug testing results.

While short-term ex vivo sensitivity testing has previously been shown to reflect clinical response in selected settings, including prior work from our group demonstrating this association in both pediatric and adult tumors^11,48^, scenarios are demonstrated in which acute metabolic responses do not translate into sustained growth control. In the clinically annotated case presented here, an apparent short-term ATP-based ex vivo response to MEK inhibition did not translate sustained clinical benefit observed in the patient whereas durable growth suppression was not observed through long-term imaging. This example illustrates how integrating conceptually distinct functional assays may improve the interpretation of drug response signals and better align ex vivo testing results with clinically meaningful treatment outcomes.

Our findings further demonstrate the feasibility of dynamic imaging across a wide spectrum of patient cancer models. Both epithelial and mesenchymal expandable long-term microtumor cultures could be analyzed, indicating that the workflow is broadly applicable and not limited to particular tumor subtypes. Replicability across samples and laboratories will be a key requirement for clinical translation, and our study demonstrates consistent performance across biologically diverse models. For fast-growing tumors, earlier endpoints may be required to avoid overgrowth in the control conditions, while more indolent models benefit from extended monitoring. This flexibility underlines the robustness of the assay across biological contexts^49^.

Looking forward, the next challenge will be to evaluate how measured ex vivo responses translate into clinical benefit. The clinically annotated case presented in this study illustrates how LTI can provide additional interpretative value by distinguishing transient metabolic responses from durable growth suppression in a real clinical context. Prospective studies linking functional assay results to patient outcomes are needed to define the predictive value of cytostatic versus cytotoxic responses, to determine how combination profiles inform therapy selection, and to establish which endpoints should be prioritized in decision frameworks. Future studies integrating longitudinal ex vivo drug profiling with m/zPDX models and prospective clinical outcome data will be important to determine which response features best predict durable therapeutic benefit in patients. Addressing these questions will be critical for integrating functional assays into routine precision oncology and for advancing their regulatory acceptance.

## Methods

### Cell culture

#### Pediatric cancer models

Human patient-derived cell lines Ewing01, ERMS01, ERMS02, SecSarc01, and USarc01 were generated as previously described^11^ from primary tumor (surgery or biopsy) of patients enrolled in the INFORM program^24^. SecSarc01 was generated with cells isolated from the corresponding patient-derived xenograft (PDX) tumor. PDX tumors were dissociated into single cells, by cutting the sample into small pieces with a scalpel and pushing them through a 100 µm mesh cell strainer, and immediately seeded for screening. The characteristics of all cell line models used in this study are described in Table 1. The SJ-GBM2 cell line (RRID:CVCL_M141) was cultured in DMEM, high glucose, GlutaMAX supplemented medium (Gibco, Paisley, UK), supplemented with 10% FCS (Sigma-Aldrich, St. Louis, MO, USA) and 5 ml penicillin-streptomycin (10000 units and 10 mg/ml, respectively, Sigma-Aldrich). The BT40, INF_R_153, NB1 (RRID: CVCL_1440) and NCI-H3122 (RRID: CVCL_5160) cell lines were cultured in RPMI 1640 medium (Gibco) supplemented with 10% FCS (Sigma-Aldrich) and 5 ml penicillin-streptomycin (10000 units and 10 mg/ml, respectively, Sigma-Aldrich). The microtumor long-term cultures were kept in Tumor Stem Media (TSM)(Gibco), composed by 500 ml of Neurobasal A medium (Gibco) and 500 ml of DMEM F12 medium (Gibco) supplemented with 10 ml Hepes (1 M, Gibco), 10 ml sodium pyruvate (100 mM, Sigma-Aldrich), 10 ml of MEM non-essential amino acids’ solution (100x, Gibco), 10 ml of L-Glutamine (200 mM, Sigma-Aldrich), 10 ml penicillin-streptomycin (10000 units and 10 mg/ml, respectively, Sigma-Aldrich), 20 ml of B27 supplement (50x, Gibco), 1 ml of heparin (2 µg/mL.Sigma-Aldrich), 200 µl of human EGF (100 µg/ml, Peprotech, location), 200 µl of human FGF-basic (100 µg/ml, Peprotech) and 500 µl of human PDGF (20 µg/ml, Peprotech). All lines and models were cultured at 37 °C and 5% CO_2_ atmosphere. All models were subjected to Multiplex Cell Authentication (Multiplexion) and underwent routine testing for mycoplasma contamination.

#### Adult cancer models

Adult patient-derived long-term cultures (CRC, SA, PC) were generated and characterized as described before. In brief, human samples were obtained in accordance with the Declaration of Helsinki; liposarcoma HLS1/2 spheroids, colorectal CRC1/2 spheroids and pancreatic semi-adherent PC01 cultures were generated and cultured under serum-free conditions^50–54^ at 37 °C and 5% CO2. For microtumor generation from long-term cultures, singularized cells (~500–960 cells/well) were seeded in 384-well ultra-low attachment plates (Sbio, Hudson, NH, USA) for microtumor formation in production medium (3DTwin CM01; PreComb Therapeutics AG). All long-term cultures were subjected to Multiplex Cell Authentication (Multiplexion) and underwent routine testing for mycoplasma contamination. For microtumor generation from patient-derived xenografts (PDX), cells were freshly isolated from PDX and processed by dissecting into 1–2 mm^3^ pieces. Enzymatic digestion was done until generation of a single-cell suspension or little cell clumps using Miltenyi Kit (Miltenyi, 130-095-929). Next, dissociated cells were filtered via a 40 µm Cell Strainer (NeoLab, #352340), and seeded for microtumor generation in 384-well ultra-low attachment plates (Sbio, Hudson, NH, USA).

#### Laboratory animals

Immunodeficient male and female NOD.Cg-Prkdc^scid^Il2rg^tm1Wjl^/SzJ (NSG) mice were acquired from The Jackson Laboratory (Bar Harbor, ME, USA) and subsequently bred in the animal facilities of OncoRay, Dresden, or NCT/UCC Dresden, or DKFZ Heidelberg, respectively. Animals were housed collectively in standard individually ventilated cages containing wood chip bedding (SAFE Lignocel BK 8-15, J. Rettenmaier & Söhne GmbH + CO KG, Rosenberg, Germany), enrichment and nesting materials, along with acidified tap water and a diet provided ad libitum (autoclaved RM maintenance, V1534-300, ssniff Spezialdiäten GmbH, Soest, Germany). The room temperature was maintained between 26 °C and 28 °C, while the relative humidity was regulated between 40.0% and 60%. The light/dark cycle was adjusted to a 12 h interval, with illumination activated for 12 h and subsequently deactivated for 12 h. The light period began at 6/7 am, while the dark period started at 6/7 pm. All animals were maintained under stringent, specific pathogen-free conditions, in compliance with FELASA recommendations. Depending on the tumor model, ~1 × 10^5^ to 1 × 10^6^ dissociated tumor cells derived from 3D models or previous cryopreserved PDX passages were resuspended in a 1:1 mix of FCS-free cell culture medium and Corning® Matrigel and slowly injected subcutaneously. For expansion as serial xenografts, small (~0.3 mm³) tumor fragments were implanted subcutaneously through a small skin incision. Mice were anesthetized on a 37°C heating pad with 1.75% isoflurane in the inhaled air and preoperative analgesia and perioperative pain management were performed according to approved animal experimentation protocols. The transplanted mice were at least seven weeks old and were examined daily by certified animal care takers. Checkups included regular weight measurements. Animals were observed for up to 1 year (Heidelberg) or 78 weeks (Dresden) following xenotransplantation, unless tumor growth reached a maximum size or other termination criteria were met. The mice were euthanized via cervical dislocation and tumor tissue was collected for further analysis. PDX-derived material was used for ex vivo drug testing without prior depletion of murine stromal cells, and drug responses were evaluated based on relative treatment effects within each model. All animal experiments in this study were carefully planned, complied with national guidelines and received review and approval from an institutional review board/ethics committee overseen by the designated animal welfare officer. Experiments were approved by the responsible national authorities, specifically the Regierungspräsidium Karlsruhe, Baden-Wuerttemberg, Germany (animal permits G-2/20, G-3/20) and the state directorate of Saxony, Germany (animal permits TVV49/2018, TVV39/2023).

#### Zebrafish Line

Care and breeding of zebrafish (Danio rerio) were as described previously^55,56^ under institutional approval of the Regierungspräsidium Karlsruhe (35-9185.64), which conform to the Guide for the Care and Use of Laboratory Animals published by the US National Institute of Health (NIH Publication No. 85-23, revised 1996). The Tg (fli1:EGFP) strain of zebrafish embryos was used. Embryos were maintained in a PTU buffer: E3 buffer (292.2 mg/L NaCl; 12.6 mg/L KCl; 36.3 mg/L CaCl_2_; 39.8 mg/L MgSO_4_) supplemented with 0.2 mM 1-phenyl-2-thiourea (PTU, Sigma-Aldrich) at 28 °C before the experiment.

#### Zebrafish embryo toxicity assays

The toxicity of the drugs of interest was evaluated before conducting in vivo tumor growth experiments. The maximum tolerated dose (MTD) was defined as the concentration at which embryos exhibited no signs of toxicity, including mortality or morphological abnormalities such as body curvature, yolk sac deformation, cardiac edema, or lack of response to external stimuli. Previously, MTDs of 1 μM for selinexor and 20 μM for dactinomycin were established^57^. The MTD for the combination of selinexor (Selleckchem) and dactinomycin (MedChemExpress) was determined using a matrix toxicity assay. Briefly, pairwise combinations of the ¼ MTD, ½ MTD, or full MTD concentrations of each drug dissolved in PTU buffer were applied. Three zebrafish embryos per treatment condition, including the corresponding solvent control, were used. Treatment of zebrafish embryos started at 48 h post-fertilization (hpf) and was conducted at 34 °C in uncoated 48-well plates (Corning) until 120 hpf. Bright-field images of treated embryos were obtained at both 72 hpf and 120 hpf using the ImageXpress Micro Confocal High-Content Imaging System (Molecular Devices) equipped with a 4x objective lens. The MTD for the selinexor–dactinomycin combination was determined to be 0.5 μM selinexor and 10 μM dactinomycin.

#### Cell preparation for zebrafish embryo xenotransplantation

USarc01 and Ewing01 spheroids were dissociated into single cells using TrypLE Express (Thermo Fisher Scientific) for 5 min at 37 °C, followed by filtration through a 40 µM cell strainer (Greiner). The concentration of viable cells per milliliter was determined using the automated ViCell cell counter (Beckman). Subsequently, cells were stained with 5 µL of Vybrant™ CM-DiI Cell-Labeling Solution (Invitrogen) solution per 1 × 10⁶ viable cells for 10 min at 37 °C. After staining, cells were centrifuged and washed three times with FCS-free RPMI medium without phenol red. The labeled cells were resuspended in 1 mL of the same medium and incubated with 1 µL of Benzonase (Sigma-Aldrich) for 10 min at room temperature. Following another centrifugation step, the cells were resuspended to a final concentration of 1 × 10⁸ cells/mL. During injections, labeled cells were maintained on ice in the dark. Embryos were anesthetized with tricaine (tricaine methanesulfonate, 0.02% w/v; Sigma-Aldrich) for 10 min and embedded in 1% low-gelling temperature agarose (Sigma-Aldrich) on microscope slides (Epredia). Approximately 10 µL of the cell suspension was loaded into glass zebrafish injection pipettes (BioMedical Instruments) and injected into the yolk sac of embryos using the FemtoJet express microinjector (Eppendorf) and Micromanipulator (Eppendorf). Tumor cell-injected embryos were maintained at 34 °C. MTDs of selinexor, dactinomycin, and their combination were administered to USarc01 zPDX and Ewing01 zPDX models. The treatment duration was 48 h, spanning from 24 h post-implantation (hpi) to 72 hpi.

#### Confocal imaging of tumor cell proliferation and analysis of treatment efficacy in zebrafish xenografts

To monitor the proliferation of fluorescently labeled tumor cells within the zebrafish host, injected embryos were imaged at 24 h post-implantation (hpi) and 72 hpi using the ImageXpress Micro Confocal High-Content Imaging System (Molecular Devices). Images were acquired across 35 focal planes using a 4x objective lens. The treatment response to tumor growth was assessed by measuring changes in tumor volume from baseline (24 hpi to 72 hpi). Tumor volume was determined using an in-house macro developed for FIJI software, as described previously^18,56^. The best treatment response was evaluated according to the Response Evaluation Criteria in Solid Tumors (RECIST) version 1.1 modified for zebrafish xenografts. Specifically, progressive disease (PD) was defined as at least a 20% increase in tumor volume; stable disease (SD) as a change between a 20% increase and a 30% decrease in tumor volume; and partial response (PR) as at least a 30% decrease in tumor volume.

#### Short-term drug sensitivity profiling (DSP)

Pre-spotted 384-well ultra-low attachment drug plates (Corning) were used for the 72-hour endpoint drug sensitivity testing of pediatric and adult tumors, containing drugs (added randomized), DMSO/Water-Tween (negative control, adult, Dresden), DMSO (negative control, pediatric, Heidelberg), benzethonium chloride and staurosporine (positive controls). Drugs were tested in duplicates at five different concentrations. After adding cell suspension, the final DMSO/Water-Tween concentration was 0.5% (adult, -Dresden) or 0.1% (pediatric, Heidelberg). For the drug response assay, short- or long-term cultures were dissociated to single cells (adult) or freshly isolated PDX tumor cell isolates or long-term cultures of patient tumors (pediatric), counted and seeded at densities ranging from 500 to 2000 cells/well in Testing Medium (MFTM, NCT-Dresden; Supplementary Table 1) or TSM complete (pediatric, KiTZ-Heidelberg)^11^ onto the pre-spotted drug plates. Fresh-tissue drug screening (case report) was performed on heterogeneous single-cell suspensions derived from patient tumor material without prior tumor cell purification, reflecting the cellular composition of freshly processed clinical samples. Three days post seeding, treatment effect was quantified as metabolic activity readout using Cell Titer Glo 2.0 (Promega, order #G9242) according to manufacturer’s instructions. Individual drug sensitivity was evaluated using the R/Shiny application iTreX^16^, the Drug Sensitivity Score (DSS_asym_), quality control (QC) and sensitivity criteria (SC) plus quantile, as previously described^11,16^.

#### Quality control and replication of drug screening assays

Quality control of the drug screening assays was ensured through the inclusion of positive controls (staurosporine, benzethonium chloride), negative controls (DMSO and media-only), and a panel of reference chemotherapeutic agents (doxorubicin, gemcitabine, olaparib, oxaliplatin, paclitaxel) in each screening plate. All models were screened using two independent drug plate layouts, generating separate microtumor plates to assess inter-plate reproducibility. Control responses and reference drug effects were compared across plates to confirm assay sensitivity, dynamic range, and consistency of drug response profiles. Experiments included both biological and technical replication: biological replicates consisted of independent screening runs per model, while technical replicates included 12 wells per plate for negative controls and triplicate wells for each drug condition. This design enabled robust normalization, assessment of baseline variability, and evaluation of reproducibility across independent experiments. Quality control for short-term CTG assays was performed as previously described^11^.

#### Long-term dynamic drug response testing

Microtumors were produced by seeding 500 to 1540 cells (seeding numbers in Supplementary Data 1) into each well of a 384-well ultra-low attachment U-bottom plate (Sbio, Hudson, NH, USA). The plate was incubated at 37 °C and 5% CO_2_ for 72 h until full microtumor formation. Drug plates were received from FIMM (Biomedicum Helsinki, Helsinki, Finland). Each drug on the drug source plate was plated to achieve drug-specific C_max_ values for each individual drug in the test plates (full drug list in Supplementary Data 1). Staurosporine (1 µM) was used as a positive control, and 0.5% DMSO and aqua were used as negative controls. Microtumors were exposed to drugs for 24–96h h, after which the drug-containing medium was replaced with fresh medium. Drug dosing was carried out by removing 30 µl (half of the well volume) and replacing it with the corresponding treatment solution prepared at 2x concentration, ensuring the desired final testing concentration in each well. The treatments were incubated at 37 °C and 5% CO2 for 24 h or 4 days with redosing. Microtumor growth was monitored longitudinally for up to 14 days to assess the downstream effects of transient pathway perturbation on long-term growth dynamics and regrowth behavior. Each condition was done in triplicate, and the negative controls were represented in 12 wells. Upon the end of the incubation time, the treatments were removed by performing a medium change. The first medium change for each treatment was the drug removal, consequently, the medium was changed each 2/3 days.

#### Response analysis

Bright field images were acquired every 2–3 days during 14 days at 37 °C using an ImageXpress Micro Confocal (Molecular Devices, San Jose, CA, USA) high-content microscope. The produced images were then processed through an automated data analysis pipeline (PreComb Therapeutics, Switzerland). Briefly, the images from each well and time point were run through the tool for image segmentation, the microtumors were identified, and their characteristics recorded for consecutive analysis. Several numerical features were extracted for each microtumor, for this study, the diameter equivalent was selected for further use. This parameter is calculated as if the microtumor would be a perfect sphere of the same volume as the one reconstructed after corrections considering all the parameters identified for that specific condition, well and time point. This information was pooled together for each well and a size progression curve was plotted. The treatment-to-control ratio (TC) was computed to further rank the performance of the treatments in the different cell line models.

#### Automated image segmentation

Brightfield microscopy images were automatically segmented using a deep learning–based semantic segmentation approach. Images were segmented into microtumor and background regions using a convolutional neural network (CNN). The model outputs binary segmentation masks, from which connected components corresponding to individual microtumor tissues were extracted and quantified. The neural network was trained on 2308 expertly annotated images and evaluated on an independent test set of 571 images, achieving an Intersection over Union (IoU) of 0.92. IoU was calculated as the ratio of the area of overlap to the area of union between predicted and ground-truth segmentation masks. A subset of inference masks was manually reviewed and corrected by domain experts to ensure segmentation accuracy.

#### Data analysis

The area (A) and perimeter (P) of each 3D tumor model piece are calculated based on the segmented mask. Then, the volume of the spheroid can be approximated as a product of the simple volume (V) equivalent of a sphere of such area (Eq.1) and a sphericity correction factor (φ) (Eq.2)^58^. The resulted corrected volume (V’) (Eq.3) is then transformed to an equivalent diameter (d) (Eq.4) to operate with linear units. In a case of multiple objects in the well, first the total volume of all the 3DTwins is calculated and then one equivalent diameter is derived. Progression of the diameter d over time is then scaled to the last time point before treatment (“day zero”) for each individual well. Thus, relative size (d’) progression of every 3D tumor model starts at 1 at day zero and grows or shrinks to (1 + Δd’) over the 14 days assay window. After grouping the data points of the same treatment condition of each plate, we fit a 3^rd^ degree polynomial to the data and calculate the area under the curve (AUC) and the cumulative standard deviation of the fitted line (dAUC) for the time window from 0 to number of days in the assay. Treatment to control ratio (TC) is a ratio of AUC of each treatment condition (AUC_T_) to the AUC of a respective negative control (AUC_C_) (from the same plate). To derive a standard deviation of TC function (dTC) one should use a law of propagation of uncertainty^59^, which for a ratio of two variables can be formulated through the relative uncertainty: To rank the treatments by their efficacies, one can directly use the TC values (accompanied by the dTC variability to consider the significance of such responses). Alternatively, one can pursue the classical RECIST criteria and compare each progression to its own starting point. We categorize responses by comparing the AUC_T_ to the area of a trapezium from 1 to (1 + Δd’) over the assay time window (assuming the equivalent diameter d grows linearly, as well as the relative size d’ used for AUC_T_ calculation). The thresholds on Δd’ used in the analysis: +20% and more - progressive disease (PD), ±20% - stable disease (SD), from -20% to -50% - Partial remission (PR), -50% and more - Strong remission (SR).

#### ROC analysis and data visualization

Receiver operating characteristic (ROC) analysis was performed to evaluate the ability of the long-term imaging (LTI) score to discriminate genotype-matched from non-matched drug–model pairs in the BRAF^V600E^ BT40 model. The binary match annotation (matched = 1, non-matched = 0) was used as reference. ROC curves were generated by varying LTI score thresholds and calculating sensitivity and 1−specificity. The area under the curve (AUC) was computed using the trapezoidal rule, and the optimal threshold was determined using the Youden index (sensitivity + specificity − 1). In addition, sensitivity and specificity were reported for a predefined cutoff of LTI ≥ 0.7 across positive control models.

Overlap of drugs across assay platforms (STA_ped_ vs. LTI and STA_adu_ vs. LTI) was visualized using UpSet-style plots. Drugs were categorized as platform-specific or shared, and counts were displayed as stacked bars distinguishing chemotherapeutic and targeted agents. A dot matrix indicated platform membership and shared subsets.

The temporal structure of drug exposure was visualized using segmented lollipop plots. Treatment duration was represented as the initial segment of each timeline, followed by the drug-free monitoring phase up to the total assay duration.

Concordance between STA results, imaging-based RECIST-like response categories, and response strength was visualized using Sankey diagrams. STA top hits (patient quantile rank >75%, *n* = 40) were mapped to RECIST-like categories (CR, PR, SD, PD) and subsequently to response strength classes (strong, weak, non-responder) derived from control-normalized AUC values.

#### Fusion detection from RNA sequencing data

Gene fusion detection was performed using Arriba (v2.2). RNA-seq reads were aligned to the human reference genome using STAR in chimeric-read detection mode to enable identification of split and discordant alignments indicative of fusion events. The resulting chimeric junction and alignment files were processed with Arriba to call candidate fusion transcripts. Default Arriba filtering parameters were applied to remove likely artifacts, including read-through events, low-support calls, and recurrent false-positive fusions. Remaining candidate fusions were manually inspected using Arriba’s visualization output to confirm breakpoint consistency and sufficient supporting read evidence. High-confidence fusion events were reported for each INFORM cell line sample and, where applicable, cross-validated against known oncogenic driver alterations.

#### TSNE and CNA

The molecular diagnosis, methylation profile, and copy number aberrations for patient-derived samples were assessed with the Infinium MethylationEPIC Bead Chip (Illumina) according to the manufacturer’s instructions. The resulting data for the original fresh-frozen tumors and the corresponding LTC models were used for molecular classification by comparison with reference set for sarcomas^60^ and in-house control set (healthy bone cells, healthy muscle cells and fibroblasts) via in-house application. Data was visualized in R-studio (ver. 2025.02) using the following packages: “tidyverse”, “ggplot2”, “dplyr”, “ggpubr”, “ggrepel”, “openxlsx”.

#### Statistical analysis

All statistical analyses were performed with the software program R (R version 4.4.1; The R Foundation for Statistical Computing) or GraphPad Prism version 10.0.0 for Windows, GraphPad Software, Boston, Massachusetts USA, www.graphpad.com. Statistical tests for Pearson correlation were performed using the cor.test and the method = “pearson”. For Bland-Altman plots, the packages “ggplot2”, “bestNormalize”, “tidyr”, “dplyr” and “blandr”, version 0.5.1, was used. Heatmaps (unsupervised hierarchical clustering) were calculated with the packages pheatmap_1.0.12, dplyr_1.0.9, and readxl_1.4.1. For some visualizations Python 3.14 was applied.

#### Written informed consent statement and ethical approval

The study was conducted in accordance with Good Clinical Practice guidelines and the Declaration of Helsinki. All patients, their legally acceptable representatives, or both (if possible) provided written informed consent. Approval for the study protocol (and any modifications thereof) was obtained from independent ethics committees and the institutional review board at each participating center. The INFORM program was registered with the German Clinical Trial Register, number DRKS00007623. Adult human cancer samples (male and female patients) were obtained from University Hospital Carl Gustav Carus Dresden (MASTER Dresden EK431102015) and University Hospital Heidelberg (MASTER Heidelberg S-206/2011) in accordance with the Declaration of Helsinki. Informed consent was received from each patient, as approved by the University Ethics Review Board.

## Supplementary information

Supplementary Information

Supplementary Data1

## Data Availability

Processed drug response matrices, analysis subsets, and response classifications are provided as Supplementary Data files. Custom analysis scripts used to generate the reported summary statistics are available in as Supplementary Data. The image segmentation and analysis pipeline used in this study is implemented using proprietary software developed by PreComb. The core algorithms include a convolutional neural network (CNN) for semantic segmentation and downstream image-processing routines for microtumor identification and quantification. Due to intellectual property restrictions, the full source code cannot be publicly released. However, the computational workflow and model architecture are described in sufficient detail in the Methods section to allow reproduction of the analysis.
