# Supplementary material for: Integration and validation of complementary ex vivo assays for functional precision oncology
Source: NPJ Precis Oncol. 2026 Jun 17;10:230. doi: 10.1038/s41698-026-01555-2 (PMC13275902; doi:10.1038/s41698-026-01555-2)
Supplement: Supplementary file 1 — Supplementary Information [file 41698_2026_1555_MOESM1_ESM.pdf]

Supplementary Figures and Legends

Suppl. Figure 1

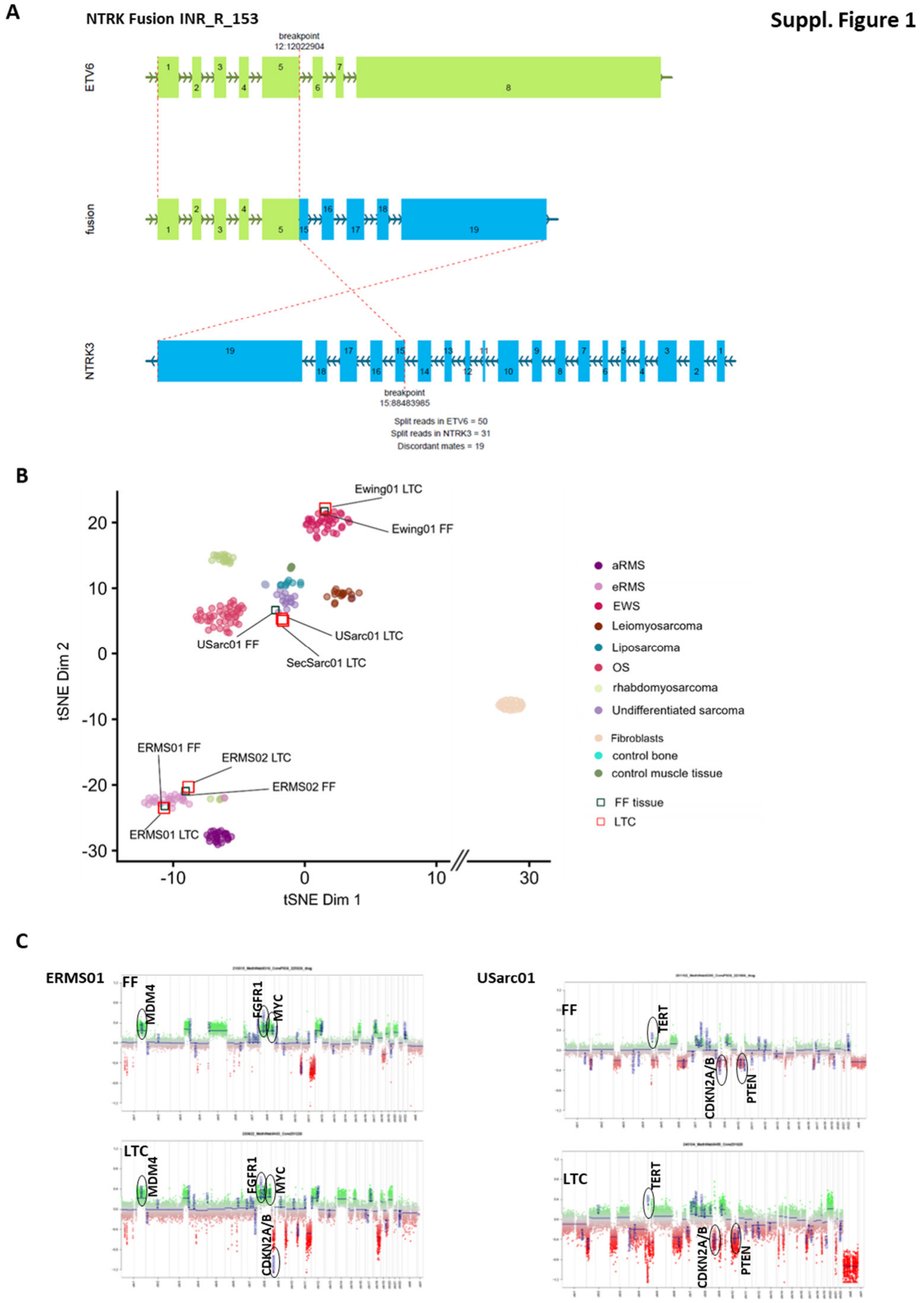

Suppl. Figure 1: Fusion detection and molecular characterization. (A) *Detection of gene fusions from RNA-seq data. RNA-seq reads from INFORM samples were aligned to the human reference genome using STAR in chimeric-read mode. Chimeric junctions and alignments were analyzed with Arriba v2.2 to identify candidate fusion transcripts. Arriba's default filtering was applied to remove likely artifacts, including read-through events, low-support calls, and recurrent false positives. Remaining candidates were manually inspected using Arriba's visualization output to confirm breakpoint consistency and supporting reads. High-confidence fusions were reported for each sample and, where applicable, cross-referenced with known oncogenic driver events.* ***(B)*** *t-SNE analysis of DNA methylation profiles for comparison of the original tumors and the corresponding tumor-derived long-term culture (LTC) models with already existing well-characterized reference tumors.* ***(C)*** *Pairwise comparison of the copy-number profiles of tumors (upper panel) and their corresponding LTCs (lower panel) reveal maintenance of relevant driver events. FF fresh frozen material of the original tumor, LTC long-term culture.*

Suppl. Figure 2

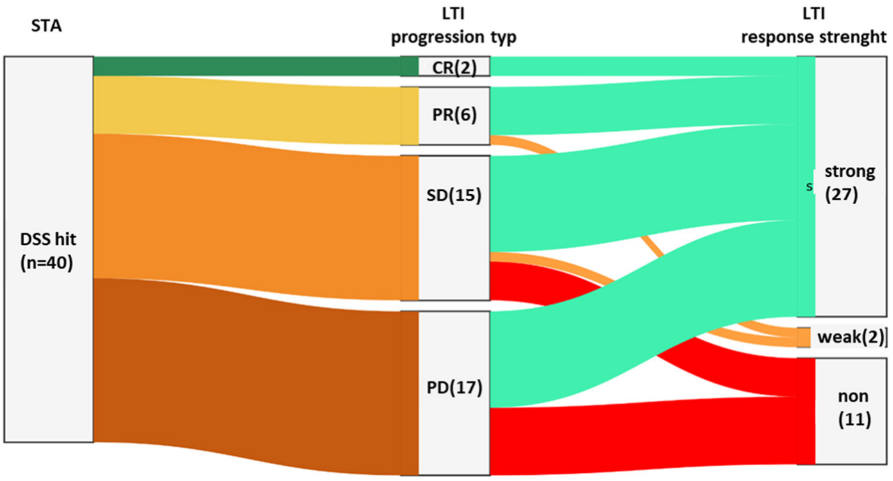

Suppl. Figure 2: Assay concordance. *Concordance between short-term CTG assay (STA), long-term dynamic imaging (LTI)-based RECIST-like classification, and LTI response strength. Sankey diagram illustrating the relationship between STA top hits (n = 40; defined by DSS patient quantile), imaging-based progression type using RECIST-like response categories (CR, complete response; PR, partial response; SD, stable disease; PD, progressive disease), and response strength normalized to solvent control (strong, weak, non-responder). Flow width is proportional to the number of drug-model pairs transitioning between categories. Absolute counts are indicated within nodes.*

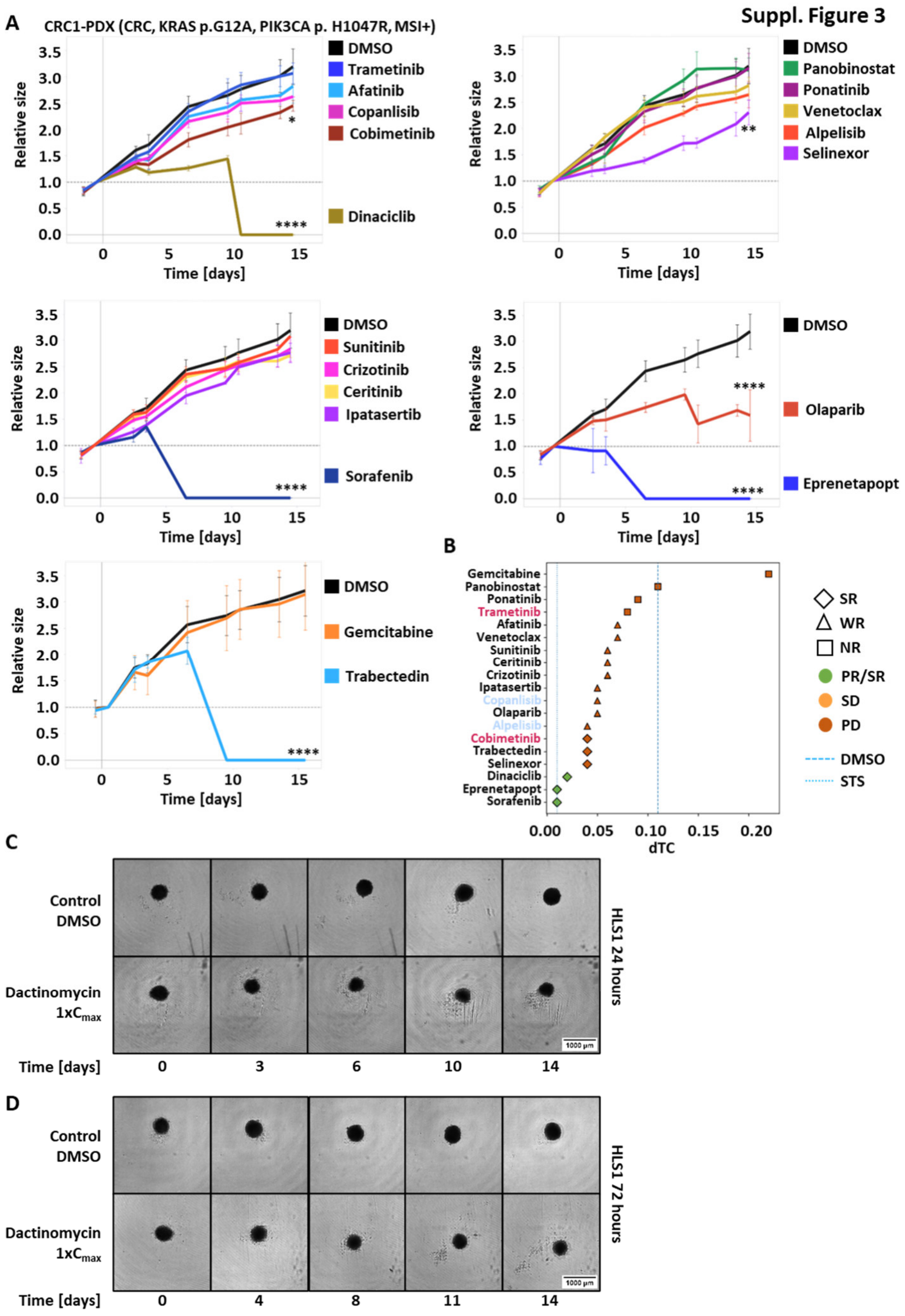

Suppl. Figure 3: Drug response dynamics in adult cancer models. (A) *Long-term dynamic imaging of the CRC1 colorectal cancer model (CRC; KRAS p.G12A, PIK3CA p.H1047R, MSI+) under treatment with a panel of targeted agents and chemotherapeutics. Relative model size was monitored over time and normalized to baseline. Drugs include inhibitors targeting MAPK signaling (e.g., trametinib, cobimetinib), PI3K/AKT signaling (e.g., alpelisib, copanlisib, ipatasertib), cell-cycle regulators (dinaciclib), nuclear export (selinexor), DNA damage response (olaparib), and additional targeted agents or cytotoxic compounds. DMSO served as the solvent control. Curves illustrate heterogeneous drug response dynamics across treatment conditions.* The relative growth shows the fold-increase in relative size (diameter) over 14 days. Error bars represent SD. Statistics: Last time point comparison to DMSO. One-way ANOVA followed by Dunnett's multiple comparisons test. * $p < 0.05$, ** $p < 0.01$, *** $p < 0.001$, **** $p < 0.0001$ ***(B)*** *Relationship between drug response classification and dynamic treatment coefficient. (dTC). Dot plot showing dTC values for the tested drugs. Marker shape indicates response type (diamond: strong responder, SR; triangle: weak responde, WRr; square: non-responder, NR), while marker color reflects imaging-based RECIST-like progression categories (green: partial remission, PR; orange: stable disease, SD; brown: progressive disease, PD). Vertical dashed and dotted lines indicate reference values for solvent control DMSO and death-control staurosporine (STS, 1 µM), respectively.* ***(C–D)*** *Representative images illustrating treatment-duration–dependent responses of HLS1 tumoroids, derived from a 73-year-old male patient with*

*differentiated liposarcoma, to dactinomycin (1× Cmax) treatment. Tumoroids were exposed to dactinomycin for 24 h **(C)** or 72 h **(D)**, followed by longitudinal imaging over 14 days to monitor growth dynamics. DMSO served as the solvent control. Scale bar, 1000 µm.*

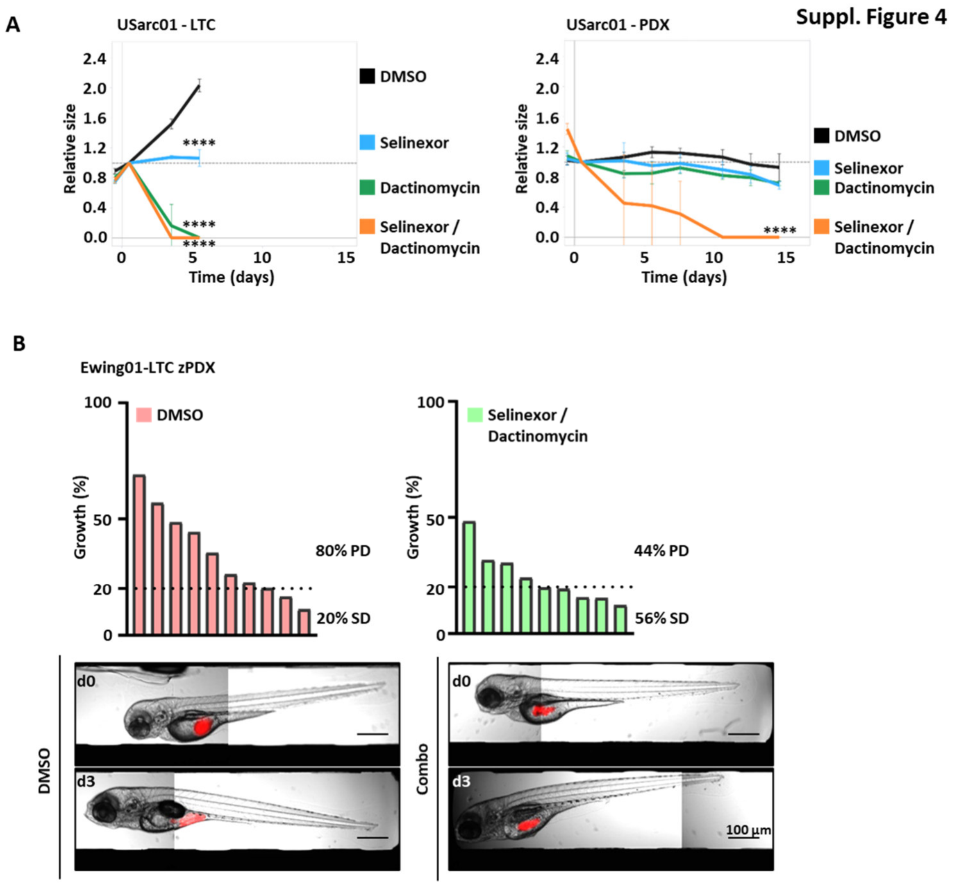

Suppl. Figure 4: Combinatorial activity of Selinexor and Dactinomycin across complementary sarcoma models. ***(A)*** *Left: LTI of the USarc01 long-term culture (LTC) model treated with selinexor, dactinomycin, or the selinexor/sactinomycin combination. Relative model size was monitored over time and normalized to baseline. DMSO served as the solvent control. The combination treatment demonstrates enhanced growth inhibition compared with single-agent treatments. Right: LTI analysis of a USarc01 patient-derived xenograft (PDX)–derived model treated with selinexor, dactinomycin, or the selinexor/dactinomycin combination, showing comparable combinatorial effects across model systems. The relative growth shows the fold-increase in relative size (diameter) over 14 days. Error bars represent SD. Statistics: Last time point comparison to DMSO. One-way ANOVA followed by Dunnett's multiple comparisons test. **** $p < 0.0001$.* ***(B)*** *Evaluation of the selinexor/dactinomycin combination in a zebrafish embryo xenograft (zPDX) model generated from the Ewing01 LTC model. Tumor growth was quantified after treatment and categorized as stable disease (SD) or progressive disease (PD). The combination treatment (selinexor 1 µM, dactinomycin 20 µM) resulted in an increased proportion of SD responses compared with control conditions. Representative microscopy images at the indicated time points illustrate treatment-associated differences in tumor burden. Scale bar, 100 µm.*

Suppl. Figure 5

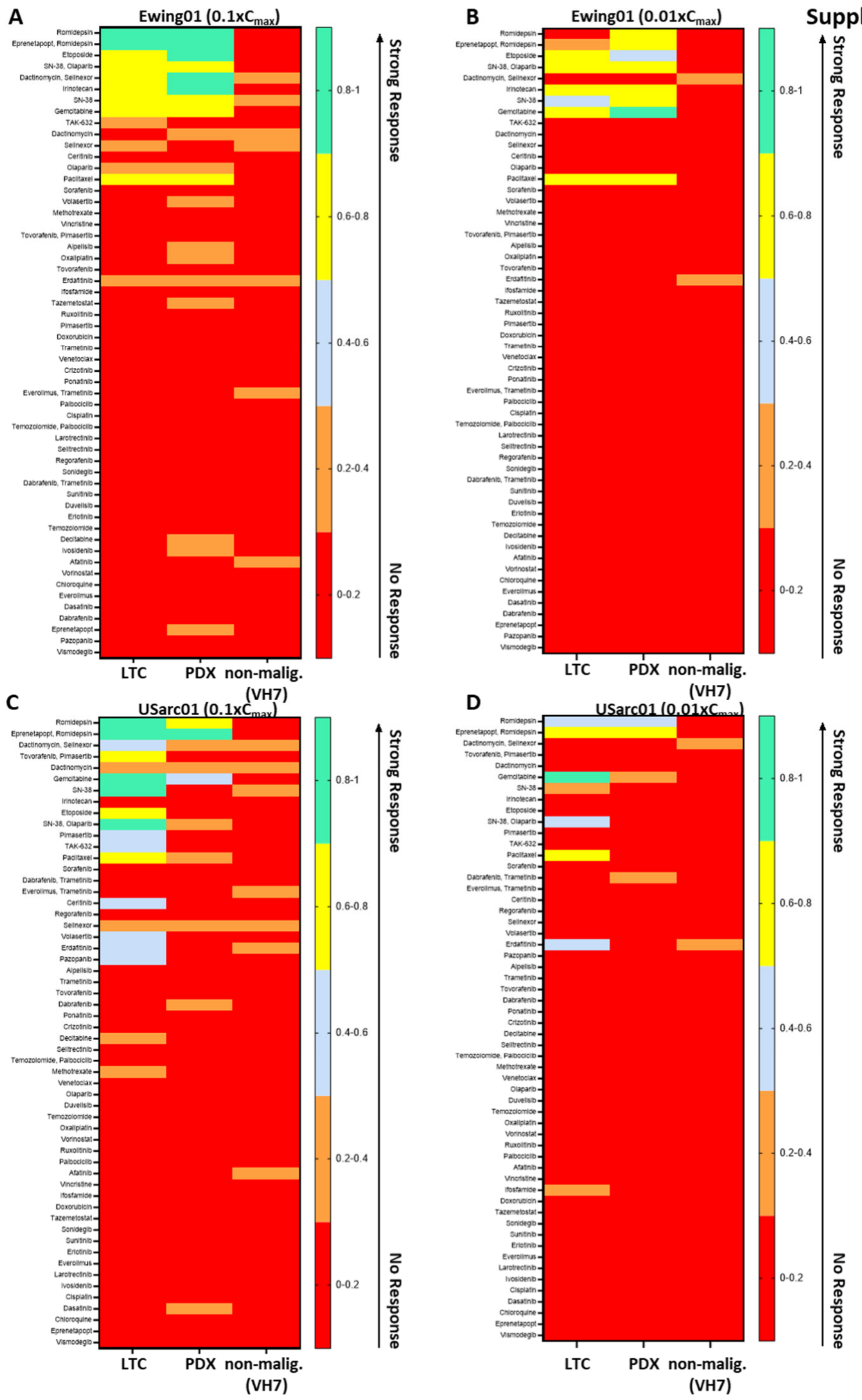

Suppl. Figure 5: Drug responses in the pediatric patient-derived models across PDX and LTC platforms. *Heatmaps summarizing the drug responses observed in the patient-derived Ewing01 (**A,B**) or USarc01 (**C,D**) model using long-term cultures (LTC) or PDX-derived cells (PDX) in comparison to non-malignant control cells (VH7). For each compound, response type at 0.1×C_{max} (**A,C**) and 0.01xC_{max} (**B,D**) is classified in five categories from strong responders (green) to no response (red).*

Suppl. Figure 6

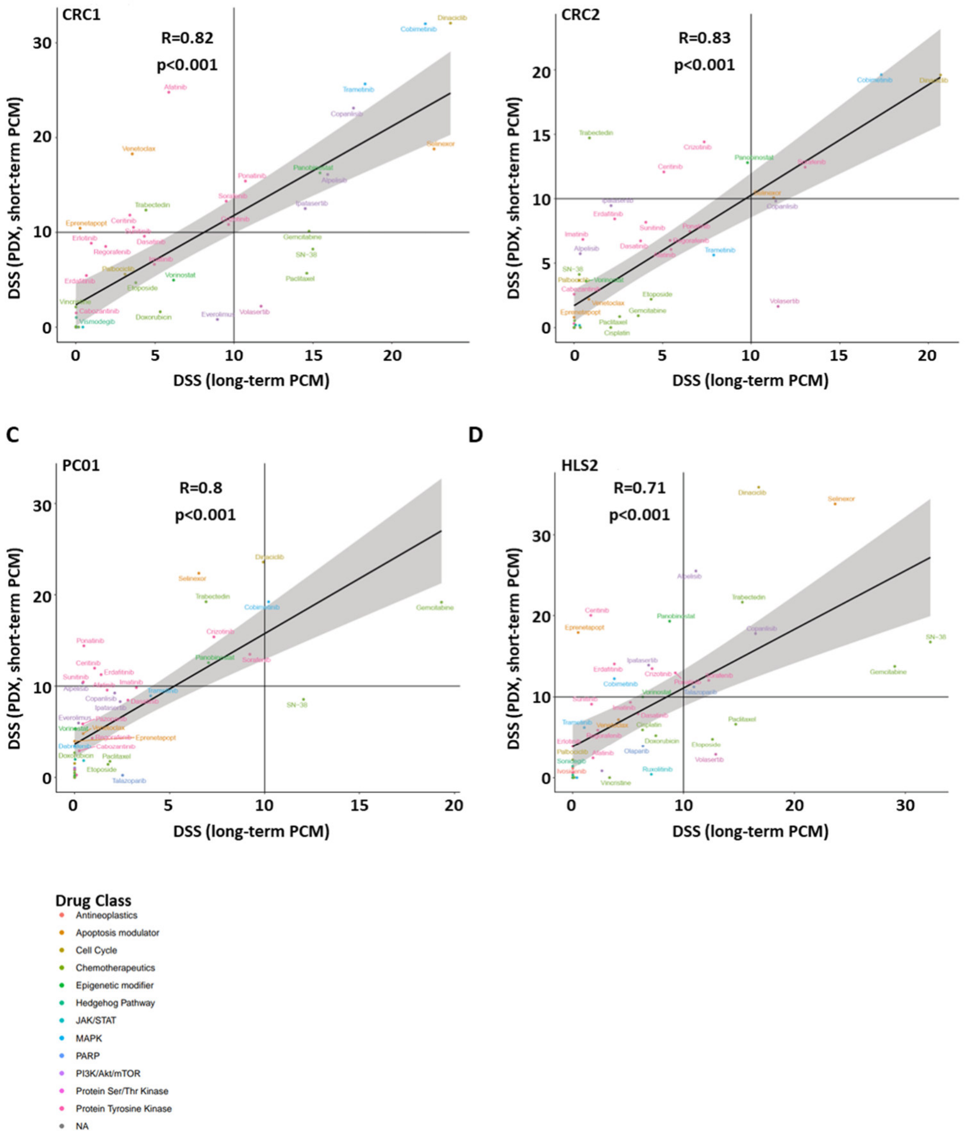

Suppl. Figure 6: *DSS$_{asym}$ Correlation between established long-term cultures and the corresponding PDX models treated across a panel of small-molecule inhibitors. Depicted are models derived from adult colorectal cancer patients CRC1 (**A**), CRC2 (**B**), pancreatic cancer patient PC01 (**C**) and myxoid liposarcoma patient HLS2 (**D**). The black diagonals represent the identity line starting at 0. Colored labels indicate the drug class of the specific drug. Spearman's rank correlation coefficient (R) is displayed.*

Supplementary Tables

Supplementary Table 1 Screening Medium NCT

Ingredients	Final Concentration in Medium	Supplier	Article Number
DMEM Advanced F12		Life Technologies	12634010
HEPES	10mM	Sigma	H0887-100mL
MEM Non-Essential Amino Acids Solution	1x	Life Technologies	11140035
L-Glutamin Solution	3,25mM	Life Technologies	25030024
B27 supplement, minus Vit. A	1x	Life Technologies	12587010
Heparin	2ng/mL	Sigma	H3149-50KU
Pen/Strep	10 U/mL	Life Technologies	15140122
hEGF	20ng/mL	R&D	236-EG-01M
hFGF basic	20ng/mL	R&D	233-FB-500/CF
PDGF-AA	10ng/mL	PeproTech	100-13A

Supplementary Table 2

model name	drug name of CTG top hit[1]	ex vivo RECIST-like[2]	response type[2] $1xC_{max}$	effective drug hit[2]	assay time (days)	treatment time (h)
BT40 ($BRAF^{V600E}$)	chloroquine	PD	NA	no	14	96
	dabrafenib	SD	strong	yes	14	96
	trametinib	SD	strong	yes	14	96
ERMS01_LTC	irinotecan	CR	strong	yes	15	24
ERMS01_PDX	selinexor	NA	strong	yes	15	24
Ewing01_LTC	olaparib	PD	strong	yes	15	24
	ceritinib	PD	strong	yes	15	24
INF_R_1288	sorafenib	PD	strong	yes	4	96
	sorafenib	PD	strong	yes	8	24
INF_R_153 ($NTRK_{fusion}$)	larotrectinib	PR	strong	yes	15	96
	ruxolitinib	PD	non	no	15	96
	selitrectinib	SD	strong	yes	15	96
INF_R_359 ($PI3K_{mut}$)	alpelisib	SD	strong	yes	14	96
	selinexor	SD	weak	yes	14	96
	dasatinib	PD	NA	no	14	96
NB1 (ALK_{amp})	ceritinib	PR	strong	yes	14	96
	crizotinib	PR	strong	yes	14	96
	ruxolitinib	PD	non	no	14	96
	volasertib	SD	strong	yes	14	96
	SN-38	CR	strong	yes	14	96
	selitrectinib	SD	strong	yes	14	96
NCI-H3122 (ALK_{fusion})	volasertib	SD	strong	yes	11	96
	ceritinib	PR	strong	yes	11	96
	crizotinib	PR	strong	yes	11	96
	palbociclib	PD	non	no	11	96
SJGBM2 (MET_{amp})	crizotinib	PD	strong	yes	11	96
	gemcitabine	PD	strong	yes	11	96
	temozolomide	PD	strong	yes	11	96
	paclitaxel	SD	strong	yes	11	96
	palbociclib	PD	strong	yes	11	96
	cisplatin	PD	non	no	11	96
	volasertib	PD	strong	yes	11	96
	decitabine	PD	non	no	11	96
	irinotecan	SD	strong	yes	11	96
SU-DIPG-25	volasertib	NA	strong	yes	14	96
	palbociclib	PD	non	no	14	96
USarc01_LTC	irinotecan	SD	strong	yes	6	24
	ceritinib	PD	strong	yes	6	24
	venetoclax	PD	NA	no	6	24
	ruxolitinib	PD	non	no	6	24
USarc01_PDX	tazemetostat	SD	non	yes	15	24
	chloroquine	SD	non	yes	15	24
UW228-2	everolimus	PR	weak	yes	14	96
	chloroquine	SD	non	yes	14	96
VH7 (non-malignant)	dasatinib	SD	non	yes	14	96
**SUM of hits**	**45**			**35**		

1) DKFZ 72h CTG metabolic activity screen
2) dynamic imaging screen
Abbreviations: PD, progressive disease; SD, stable disease; PR, partial response; CR, complete response; NA, not available.

Supplementary Table 3

Model type	Source material	Generation / processing	Culture format	Assays applied	TCC	Figures
**Primary pediatric patient-derived fresh tissue culture (FTC) – source for all ped. patient-derived models (LTC, PDX, PDX-LTC)[1]**	Fresh tumor tissue obtained from surgery or biopsy through the INFORM program	Mechanical and enzymatic dissociation	3D, FCS-free	STA, LTI	> 70%	Fig. 6
**PDX-derived fresh tissue culture (FTC)**	Fresh tumor tissue obtained from established PDX tumors	Tumor dissociation from PDX material	3D, FCS-free	STA, LTI	na	Fig. 4
**Long-term culture (LTC)**	Patient-derived fresh tumor tissue passaged at least 6 times	Expansion of cells from patient-derived FTC	3D, FCS-free	STA, LTI	90-100%	Fig. 3<br>Fig. 4<br>Fig. 5
**PDX-LTC**	mPDX-derived fresh tumor tissue passaged at least 6 times	Expansion of cells from PDX-derived FTC	3D, FCS-free	STA, LTI	90-100%	Fig. 4<br>Fig. 5<br>Fig. 6
**Molecular positive control models**	LTCs or established cell lines	Standard cell line expansion	3D<br>• FCS-free (LTC)<br>• with FCS (cell lines)	STA, LTI	90-100%	Fig. 2<br>Fig. 3<br>Fig. 5
**Non-malignant control (VH7)**	Human juvenile fibroblast line	Standard cell line expansion	3D, FCS-free	ST-CTG, LTI	0%	Fig. 2<br>Fig. 4
**Clinically annotated case model (FTC, PDX-FTC, PDX-LTC)**	Fresh tumor tissue	Mechanical and enzymatic dissociation	3D, FCS-free	ST-CTG, LTI	SecSarc01_FTC: NA<br>SecSarc01_PDX: NA<br>SecSarc01_LTC: 94%	Fig. 6
**Primary adult patient-derived long-term patient cancer models (PCM)[2-5]**	Patient tumor tissue	Mechanical and enzymatic dissociation prior to seeding into culture wells	3D, FCS-free	STA, LTI	>98%	Suppl. Fig. 3; Suppl. Fig 6
**PDX-generated from primary adult patient-derived long-term PCM**	Subcutaneous mouse xenografts tumors derived from long-term PCM	Mechanical and enzymatic dissociation of xenograft tissue prior to seeding into in vitro assays	3D, FCS-free	STA, LTI	N/A	Fig. 3, Suppl. Fig. 3; Suppl. Fig 6

STA, short-term CTG assay; LTI, long-term dynamic imaging. Culture format, exposure time, and readout parameters are detailed in the Methods section.

References:

1 Peterziel, H. *et al.* Drug sensitivity profiling of 3D tumor tissue cultures in the pediatric precision oncology program INFORM. *NPJ Precis Oncol* **6**, 94 (2022). https://doi.org/10.1038/s41698-022-00335-y

2 Dieter, S. M. *et al.* Distinct types of tumor-initiating cells form human colon cancer tumors and metastases. *Cell Stem Cell* **9**, 357–365 (2011). https://doi.org/10.1016/j.stem.2011.08.010

3 Crespo, E. *et al.* RevCAR-mediated T-cell response against PD-L1-expressing cells turns suppression into activation. *NPJ Precis Oncol* **9**, 42 (2025). https://doi.org/10.1038/s41698-025-00828-6

4 Dieter, S. M. *et al.* Patient-derived xenografts of gastrointestinal cancers are susceptible to rapid and delayed B-lymphoproliferation. *Int J Cancer* **140**, 1356–1363 (2017). https://doi.org/10.1002/ijc.30561

5 Ehrenberg, K. R. *et al.* Systematic Generation of Patient-Derived Tumor Models in Pancreatic Cancer. *Cells* **8** (2019). https://doi.org/10.3390/cells8020142

Supplementary Table 4

Combination	Tumor context	Mechanistic rationale	Key evidence
**Dabrafenib + Trametinib**	Brain tumors (high), sarcoma (limited)	Dual inhibition of MAPK pathway in BRAF V600E-driven tumors	ROAR trial : Wen et al., 2022[1]; Subbiah et al., 2023[2]
**Dactinomycin + Selinexor**	Brain tumors (moderate), sarcoma (low)	XPO1 inhibition enhances stress signaling and NF-κB suppression, potentially sensitizing to cytotoxic agents	DeSisto et al., 2020[3]
**Everolimus + Trametinib**	Brain tumors (moderate–high), CRC (low)	Combined MAPK and PI3K/mTOR pathway blockade to overcome pathway redundancy and resistance	PNOC021 (NCT04485559); Tolcher et al., 2015[4]
**Olaparib + SN-38**	Sarcoma (high), CRC (moderate)	PARP inhibition potentiates irinotecan-induced DNA damage via impaired DNA repair	Engert et al., 2015[5]; Tahara et al., 2014[6]
**Palbociclib + Temozolomide**	Brain tumors (moderate), sarcoma (indirect)	CDK4/6 inhibition enhances TMZ response and may overcome resistance mechanisms	Li et al., 2019[7]; NCT03709680
**Pimasertib + Tovorafenib**	Brain tumors (emerging), CRC (investigational)	Dual MAPK pathway targeting via MEK and RAF inhibition in MAPK-driven tumors	Rastogi et al., 2025[8]
**Carboplatin + Etoposide**	Sarcoma (moderate), CRC (low)	DNA damage induction via platinum and topoisomerase II inhibition	van Maldegem et al., 2015[9]
**Cisplatin + Doxorubicin**	Sarcoma (moderate)	Complementary DNA damage and intercalation-based cytotoxicity	Waddell et al., 1999[10]
**Dabrafenib + Trametinib**	Sarcoma (limited), CRC (moderate)	MAPK pathway inhibition in BRAF-mutant tumors; limited efficacy as doublet in CRC	Subbiah et al., 2023[2]; Corcoran et al., 2015[11]
**Everolimus + Trametinib**	CRC (low)	Combined pathway inhibition with limited clinical activity in CRC	Tolcher et al., 2015[4]
**Olaparib + SN-38**	CRC (moderate)	PARP inhibition enhances irinotecan-mediated DNA damage and replication stress	Tahara et al., 2014[6]; Chen et al., 2016[12]
**Pimasertib + Tovorafenib**	CRC (emerging)	Targeting MAPK-driven tumors via dual RAF/MEK inhibition	Rastogi et al., 2025[8]
**Temozolomide + Olaparib**	Sarcoma (high), CRC (niche)	Synthetic lethality via PARP inhibition and alkylating DNA damage	Gill et al., 2015[13]; Ingham et al., 2023[14]
**Trabectedin + Olaparib**	Sarcoma (high)	DNA damage and impaired repair via PARP inhibition in soft-tissue sarcoma	NCT02398058; NCT03838744

References:

1 Wen, P. Y. *et al.* Dabrafenib plus trametinib in patients with BRAF(V600E)-mutant low-grade and high-grade glioma (ROAR): a multicentre, open-label, single-arm, phase 2, basket trial. *Lancet Oncol* **23**, 53–64 (2022). https://doi.org/10.1016/S1470-2045(21)00578-7

2 Subbiah, V. *et al.* Dabrafenib plus trametinib in BRAFV600E-mutated rare cancers: the phase 2 ROAR trial. *Nat Med* **29**, 1103–1112 (2023). https://doi.org/10.1038/s41591-023-02321-8

3 DeSisto, J. A. *et al.* Exportin 1 Inhibition Induces Nerve Growth Factor Receptor Expression to Inhibit the NF-kappaB Pathway in Preclinical Models of Pediatric High-Grade Glioma. *Mol Cancer Ther* **19**, 540–551 (2020). https://doi.org/10.1158/1535-7163.MCT-18-1319

4 Tolcher, A. W. *et al.* A phase IB trial of the oral MEK inhibitor trametinib (GSK1120212) in combination with everolimus in patients with advanced solid tumors. *Ann Oncol* **26**, 58–64 (2015). https://doi.org/10.1093/annonc/mdu482

5 Engert, F., Schneider, C., Weibeta, L. M., Probst, M. & Fulda, S. PARP Inhibitors Sensitize Ewing Sarcoma Cells to Temozolomide-Induced Apoptosis via the Mitochondrial Pathway. *Mol Cancer Ther* **14**, 2818–2830 (2015). https://doi.org/10.1158/1535-7163.MCT-15-0587

6 Tahara, M. *et al.* The use of Olaparib (AZD2281) potentiates SN-38 cytotoxicity in colon cancer cells by indirect inhibition of Rad51-mediated repair of DNA double-strand breaks. *Mol Cancer Ther* **13**, 1170–1180 (2014). https://doi.org/10.1158/1535-7163.MCT-13-0683

7 Li, Z. *et al.* Modulating lncRNA SNHG15/CDK6/miR-627 circuit by palbociclib, overcomes temozolomide resistance and reduces M2-polarization of glioma associated microglia in glioblastoma multiforme. *Journal of experimental & clinical cancer research : CR* **38**, 380 (2019). https://doi.org/10.1186/s13046-019-1371-0

8 Rastogi, S. *et al.* Preclinical Activity of the Type II RAF Inhibitor Tovorafenib in Tumor Models Harboring Either a BRAF Fusion or an NF1 Loss-of-Function Mutation. *Cancer Res Commun* **5**, 668–679 (2025). https://doi.org/10.1158/2767-9764.CRC-24-0451

9 van Maldegem, A. M. *et al.* Etoposide and carbo-or cisplatin combination therapy in refractory or relapsed Ewing sarcoma: a large retrospective study. *Pediatr Blood Cancer* **62**, 40–44 (2015). https://doi.org/10.1002/pbc.25230

10 Waddell, A. E. *et al.* Doxorubicin-cisplatin chemotherapy for high-grade nonosteogenic sarcoma of bone. Comparison of treatment and control groups. *Can J Surg* **42**, 190–199 (1999).

11 Corcoran, R. B. *et al.* Combined BRAF and MEK Inhibition With Dabrafenib and Trametinib in BRAF V600-Mutant Colorectal Cancer. *J Clin Oncol* **33**, 4023–4031 (2015). https://doi.org/10.1200/JCO.2015.63.2471

12 Chen, E. X. *et al.* A Phase I study of olaparib and irinotecan in patients with colorectal cancer: Canadian Cancer Trials Group IND 187. *Invest New Drugs* **34**, 450–457 (2016). https://doi.org/10.1007/s10637-016-0351-x

13 Gill, S. J. *et al.* Combinations of PARP Inhibitors with Temozolomide Drive PARP1 Trapping and Apoptosis in Ewing's Sarcoma. *PLoS One* **10**, e0140988 (2015). https://doi.org/10.1371/journal.pone.0140988

14 Ingham, M. *et al.* Phase II Study of Olaparib and Temozolomide for Advanced Uterine Leiomyosarcoma (NCI Protocol 10250). *J Clin Oncol* **41**, 4154–4163 (2023). https://doi.org/10.1200/JCO.23.00402

Supplementary Data 1

Content:

KiTZ_raw results

KiTZ drug list

NCT_raw results

NCT drug list

assay treatment time

Sensitivity_Specificity

Viability at seeding

Seeding density

QC

Script ROC analysis

Script Upset plot

Script Lollipop plot

Script Sankey plot

Script Bland-Altmann

Script t-SNE
